# The nisin O cluster: species dissemination, candidate leader peptide proteases and the role of regulatory systems

**DOI:** 10.1099/mic.0.001531

**Published:** 2025-02-10

**Authors:** Jacob Scadden, Rebecca Ansorge, Stefano Romano, Andrea Telatin, Dave J. Baker, Rhiannon Evans, Cristina Gherghisan-Filip, Zhenrun J. Zhang, Melinda J. Mayer, Arjan Narbad

**Affiliations:** 1 Quadram Institute Bioscience, Norwich Research Park, Norwich, UK; 2 Earlham Institute, Norwich Research Park, Norwich, UK; 3 Duchossois Family Institute, University of Chicago, Chicago, Illinois, USA; 4 Department of Microbiology, University of Chicago, Chicago, Illinois, USA

**Keywords:** antimicrobial peptide, biosynthesis, *Lachnospiraceae*, nisin, peptidase, regulation

## Abstract

Nisin O is an antimicrobial peptide encoded by the human gut bacterium *Blautia obeum* A2-162 which has antimicrobial activity against clinically relevant organisms. The nisin O biosynthetic gene cluster (BGC) varies from other nisin BGCs as it lacks a leader-peptide cleaving protease and contains two bacterial two-component response regulator–histidine kinase (RK) systems. The dissemination of the nisin O cluster, the final proteolytic biosynthesis step and the regulation of nisin O are currently unknown and are the foci of this study. We identified six nisin O-like BGCs across *Blautia*, *Dorea* and *Ruminococcus* species using comparative genomics. These BGCs show evidence of genetic transfer between genera, with genes involved in transposition discovered up- and downstream of the BGCs. All nisin O-like BGCs contained two RK systems but no protease. Mining the *B. obeum* A2-162 genome identified candidate proteases that were cloned and used in pre-nisin O leader peptide cleavage assays. None of the candidate proteases removed the leader; however, cleavage was achieved using trypsin. To maximize the expression of the *nsoA1-4* peptides, the interactions of the two RK systems with predicted promoters in the nisin O cluster were assessed using a PepI reporter assay. We observed that the P*nsoR2K2* promoter was constitutively expressed, with NsoR1K1 increasing its activity, and that there was increased *nsoA1-4* expression when the nisin A RK system and nisin A were present. Long-read cDNA sequencing confirmed *nso* gene transcription in the heterologous expression system and identified a novel, highly expressed gene. This study provides evidence that the nisin O BGC has been transferred between different gut-associated genera, with all clusters lacking a protease and containing two RK systems. We hypothesize that this BGC has lost its protease due to negative selection as a result of high trypsin concentrations in the gut. Further work is required to maximize nisin O expression for it to be used as a potential antimicrobial therapy.

## Data Summary

Data from long-read RNA sequencing of the nisin O cluster from heterologous host *Lactococcus lactis* are available in the National Center for Biotechnology Information GenBank database under Bioproject PRJNA1156391 accession number: SRR30540219.

## Introduction

The human gut microbiome is a fiercely competitive environment, and some bacteria have evolved to produce antimicrobial products that can increase their chances of survival. One of these mechanisms is the production of bacteriocins – ribosomally synthesized peptides, either post-translationally modified or un-modified, which have antimicrobial properties [1]. A number of ecological functions of bacteriocins in the gut have been suggested, including providing niche clearance for a producer invasion, prevention of invasion by a bacteriocin-sensitive species and providing community differentiation [2]. The human gut microbiome is not the only source of these peptides, which have been found to be produced by bacteria in a range of environments, including fermented foods, the animal gut, plant systems, soil and marine environments [3,7].

Among bacteriocins, lanthipeptides are a group of post-translationally modified proteins that form highly stable lanthionine or methyllanthionine rings through the action of dehydratase and cyclase activity [8]. Some of these peptides have antimicrobial properties, termed lantibiotics, with the most studied being nisin A, produced by *Lactococcus lactis* [9]. As of 2024, there have been 12 natural variants of nisin A identified, 7 of which have been identified in the last decade [10,20]. Nisin has potential as a therapeutic agent due to its broad spectrum of antimicrobial activity against mainly Gram-positive bacteria, high pH- and temperature stability and auto-inducible expression, making nisin variants ideal candidates for further study [9]. The identification of novel antimicrobial peptides is needed in the battle against antimicrobial resistance, with nisin and other bacteriocins being potential candidates for antimicrobial therapies [21,22]. However, there is evidence that resistance to nisin and antimicrobial peptides is widespread and that prior to therapeutic use resistance mechanisms should be taken into account [23].

The nisin O gene cluster has been identified in the human gut bacterium *Blautia obeum* A2-162, a Gram-positive, non-motile, coccus to coccobacillus-shaped, obligate anaerobe [15,24]. This bacterium is found in abundance in the gut, and the genus to which it belongs has been linked with health benefits and has potential probiotic attributes [25]. Reduced levels of *Blautia* in the human gut have been associated with increased risk of colorectal cancer and type I diabetes in children, and a reduction in *Blautia* abundance has been observed in patients with Parkinson’s disease; conversely, increased levels have been related to reduced inflammation and reduced risk of graft vs. host disease [26,30]. Nisin O has shown antimicrobial activity against *Clostridium perfringens* in the presence of trypsin in *in vitro* assays [15]. The biosynthetic gene cluster (BGC) of nisin O differs from the nisin A BGC. It contains four structural peptides, an additional two-component response regulator–histidine kinase (RK) regulatory system, and does not contain a protease, nor are there any peptidase domains in the other proteins such as NsoT [15]. Commonly, cleavage of the leader peptide by NisP is required as the final step of nisin biosynthesis, as prepeptides have no antimicrobial activity [9]. However, more recently described active nisin variants do not contain classic NisP homologues: a blauticin-producing BGC from *Blautia producta* SCSK with similarity to nisin O encodes a putative signal peptidase at the start of the cluster [31], while nisin S from *Ligilactobacillus salivarius* P1CEA3 is suggested to be cleaved by a protease found elsewhere within the genome [15]. Other bacteriocins, including ruminococcin A and subtilin, also lack the leader peptide protease within their respective BGCs [32,33].

As *B. obeum* A2-162 is found in the human gut its antimicrobial peptides may be well-adapted to function in this environment and represent potential therapeutics for gastrointestinal disorders. *Blautia producta* SCSK has already shown therapeutic promise in the treatment of vancomycin-resistant *Enterococcus* and has been shown to have antimicrobial effects against a range of gut pathogens and commensals [31,34]. The study by Zhang *et al.* also tested six nisin O-like antimicrobial peptides, including nisin O itself and blauticin, using an *Escherichia coli* heterologous expression system [34,35]. This investigation clearly showed the potential of these antimicrobial peptides to kill pathogenic and commensal members of the gut microbiota. Therefore, if the nisin O BGC could be identified in other *Blautia* species, this could provide evidence of the important role nisin O plays in the survival of this organism within the complex microbial community in the human gut. Additionally, observing the dissemination of the BGC in other genera could demonstrate horizontal gene transfer (HGT) of this antimicrobial and illustrate its importance to its hosts in clearing and/or maintaining their niche. Understanding how nisin O peptides are produced, regulated and activated and how widespread this cluster is in nature has not yet been fully established. In this study, we identified similar clusters in several genera, all of which lacked a protease. Candidate proteases were identified from the *B. obeum* A2-162 genome, and their function was analysed. To investigate the regulation of the nisin O BGC, we assessed the interaction of the two nisin O regulatory systems with the putative promoters in a PepI reporter assay. Using Oxford Nanopore Technology sequencing of cDNA in the *L. lactis* heterologous expression system, we were able to identify which genes were being expressed in this system.

## Methods

### Bioinformatic searches and analysis of nisin O-like clusters

To identify whether nisin O-like BGCs are present in other bacteria, an initial search for proteins with homology to the NsoB amino acid sequence (ASY03218.1) was performed using blastp (using default search parameters) [36] (RRID:SCR_001010) with standard databases and no specific organisms selected. Results were filtered using a 95% minimum percent identity and a 60% minimum query coverage. Seven organisms identified with the protein were downloaded into Geneious R11 (v.11.1.5, Biomatters Ltd) (RRID:SCR_010519), and an annotation search was performed manually using the terms ‘nisin’ and ‘lantibiotic’, identifying a full nisin O-like BGC in each. Clusters identified were extracted, and each gene was translated and aligned to the corresponding amino acid sequence from the nisin O cluster using muscle v.3.8.425 [37] (RRID:SCR_011812).

All nisin O-like BGCs identified using the blastp analysis were found in *Lachnospiraceae*; therefore, using a large-scale comparative genomic analysis, we verified the prevalence of the nisin O-like BGC within the *Lachnospiraceae* family. Two thousand ninety-four genomes were identified as belonging to *Lachnospiraceae* on 9 March 2021 at the Genome Taxonomy Database (GTDB) (v80) [38], and respective assemblies were downloaded from the National Center for Biotechnology Information (NCBI) using ftp links retrieved with NCBImeta [39]. Genome completeness and quality were assessed using CheckM (v.1.0.18) [40] (RRID:SCR_016646) and the marker gene set for the *Lachnospiraceae* family (GTDB v.1.0.18) which excluded genomes that had <95% completeness or >5% contamination. The 1430 remaining genomes were then annotated using Prokka (v.1.14.5) [41] (RRID:SCR_014732), secondary metabolite BGCs identified using antiSMASH v.5.0 (bacterial version) [42] (RRID:SCR_022060) and clustered using BiG-SCAPE [43] (RRID:SCR_022561). OrthoFinder (v.2.5.2) [44] (RRID:SCR_017118) was then used to identify orthologues of *nisB* and *nisC* as markers for the potential presence of nisin-like BGCs within the annotated genomes. All bacterial genomes that contained orthologues of either *nisB* or *nisC* identified by the comparative genomic analysis were then analysed manually using Geneious R11 and BAGEL4 [45] to confirm the presence of the nisin O BGC. BAGEL4 was used in addition to antiSMASH as we found that antiSMASH did not identify one of the nisin O-like BGCs although both NsoB and NsoC genes were present, whereas they were found when using BAGEL4. The newly identified BGCs were then compared to verify consistency in cluster composition and synteny using the Artemis Comparison Tool (ACT) [46] (RRID:SCR_004507) and Clinker [47].

To investigate the potential HGT of these BGCs, maximum likelihood phylogenetic trees of the nisin O-like cluster nucleotide sequences and the host 16S rRNA sequences were aligned using clustal omega (v.1.2.4) and the maximum likelihood tree generated using RAxML (v.8.2.12) [48] (RRID:SCR_006086) and visualized on iTOL (v.6.8.1) [49] (RRID:SCR_018174).

### Mining the *B. obeum* A2-162 genome for candidate proteases

As other bacteriocins, such as ruminococcin A, have proteases capable of leader peptide cleavage elsewhere on their genomes, we investigated the *B. obeum* A2-162 genome (Taxid:657314) for candidate proteases using tblastn (Expect value=0.01, Gapcost=11,1, Matrix=BLOSUM62) [36], with the amino acid sequence of LanP from *B. producta* SCSK (WP_171285516.1) used as the query sequence. In addition, a conserved catalytic region of the *B. producta* SCSK LanP was identified (26_SPase_1; PF10502) using BATCH CD-Search [50] which was then used to identify similar peptidases in the *B. obeum* A2-162 genome. The *B. obeum* A2-162 genome was also searched manually using the query terms ‘subtilisin’ and ‘serine-like protease’. These query terms were selected due to known evidence that subtilisin, a serine protease, can cleave the leader peptide of subtilin [33]. Amino acid sequence alignment of the candidate proteases was performed by muscle (v.3.8.425) [37] using Geneious R11 to analyse any regions of homology compared to the known nisin proteases LanP and NisP (AAA25200.1).

### Bacterial strains, plasmids and culture conditions

Strains and plasmids are described in Table 1. All chemicals were from Sigma-Aldrich unless stated otherwise. *B. obeum* A2-162 and *C. perfringens* NCTC 3110 were grown anaerobically in Brain Heart Infusion Broth (Oxoid) with complements (50 mg l^−1^ vitamin K, 1 mg l^−1^ resazurin, 5 mg l^−1^ hemin and 0.5 g l^−1^ l-cysteine) in an anaerobic cabinet (Don Whitley, UK; 5% CO_2_, 10% H_2_ in N_2_ at 37 °C) for 24 h. *L. lactis* strains were grown at 30 °C for 16 h in M17 medium (Oxoid) with the addition of 5 g l^−1^ glucose (GM17) after autoclaving. *Escherichia coli* strains were grown in Lennox L broth (10 g l^−1^ bacto tryptone (Difco), 5 g l^−1^ bacto yeast extract (Difco), 5 g l^−1^ NaCl and 1 g l^−1^ glucose) at 37 °C with shaking at 250 rpm for 16 h. Antibiotic selection was performed using chloramphenicol and erythromycin at 5 µg ml^−1^ for *L. lactis* and 15 µg ml^−1^ and 400 µg ml^−1^ for *E. coli*, respectively, and 100 µg ml^−1^ ampicillin for *E. coli*. When inducing nisin O producing strains, 10 ng ml^−1^ nisin A was added [15]. Trypsin stocks (10 mg ml^−1^) were made using 50 mM acetic acid.

**Table 1.** Strains and plasmids used in this work

Strain	Relevant characteristics	Reference
*B. obeum* A2-162	Strain isolated from human GI tract∗	[15]
*L. lactis* MG1614	*L. lactis* subsp. *lactis* 712 containing no plasmids or prophage, no nisin genes	[84]
*L. lactis* UKLc10	Nisin A *nisRK* genes integrated on the chromosome	[55]
*E. coli* MC1022	Shuttle vector cloning strain	[85]
*E. coli* BL21 (DE3)	Chemically competent protein expression strain	Invitrogen
**Plasmid**	**Relevant characteristics**	**Reference**
pIL253 (taxonomy ID 71279)	*Lactococcus* plasmid; erythromycin resistance	[86]
p*nso*	Nisin O lantibiotic cluster (GenBank KY914474) in pIL253	[15]
pUK200	*E. coli*/*Lactococcus* shuttle vector; chloramphenicol resistance	[55]
pORI280	*L. lactis* P32 promoter fused to *lacZ;* erythromycin resistance	[51]
pET15b	Expression vector; contains N-terminal His-tag; ampicillin resistance	Novogen
pET15b_*p66*	Candidate protease *p66* in pET15b	This study
pET15b_*p570*	Candidate protease *p570* in pET15b	This study
pET15b_*lanP*	*B. producta* SCSK *lanP* in pET15b	This study
pET15b_*p140*	Candidate protease *p140* in pET15b	This study
pP*nsoA*_*pepI*	*pepI* with 581 bp predicted promotor region P*nsoA* in pIL253	[69]
pP*nsoR2K2*_*pepI*	*pepI* with 259 bp predicted promotor region P*nsoR2K2* in pIL253	[69]
pP*nsoBTC*_*pepI*	*pepI* with 408 bp predicted promotor region P*nsoBTC* in pIL253	[69]
pP*nsoFEG*_*pepI*	*pepI* with 774 bp predicted promotor region P*nsoFEG* in pIL253	[69]
pP*nisA*_*pepI*	*pepI* with the promoter region P*nisA* in pUK200	[69]
pUK200_P32_*R1K1*	*nsoR1K1* with promoter P32 in pUK200	This study
pUK200_P32_*R2K2*	*nsoR2K2* with promoter P32 in pUK200	This study
pUK200_P32_*nso1.14*	*Nso1.14* with promoter P32 in pUK200	This study

* Kindly Pprovided by Prof. Harry Flint and Dr. Sylvia Duncan, Rowett Institute, University of Aberdeen.

### Cloning of candidate proteases and regulatory systems into pUK200_P32 and pET15b

Regulatory systems were placed downstream of constitutive promoter P32 [51] using splice overlap PCR [52] with Phusion (Finnzymes) and primers which incorporated restriction sites to facilitate cloning into pUK200 (Table S1, available in the online Supplementary Material, Sigma Genosys). Restriction (New England Biolabs) and ligation (Fast-Link DNA Ligase, Epicentre) into pUK200 were performed as per the manufacturers’ instructions. Ligation products were transformed into electrocompetent *E. coli* MC1022, and confirmed plasmids were transformed into electrocompetent *L. lactis* strains as described previously [53]. Candidate proteases *p570*, *p66*, *p140* and *B. producta* SCSK *lanP* (Fig. S1 and Table S2) were cloned into pET15b downstream of the N-terminal histidine tag then transformed into chemically competent *E. coli* BL21 (DE3) (Invitrogen) for expression [53]. For plasmid maps and insertion sequence details, see Fig. S1 and Table S2.

### Induction and purification of pre-NsoA1-4 peptides

An overnight culture of *L. lactis* UKLc10 p*nso* was used to inoculate 100 ml pre-warmed GM17 containing erythromycin to an optical density at 600 nm (OD_600_) of 0.1 and grown at 30 °C. At OD_600_ 0.5–0.6, the cultures were induced with 10 ng ml^−1^ nisin A and incubated for 3 h at 30 °C, as previously described [15]. Fifty-millilitre portions were centrifuged at 1700 ***g*** for 15 min at 4 °C. The supernatants were collected and filtered using a 0.45-µm filter. Proteins were precipitated via the addition of 10 ml 100% TCA to 40 ml supernatant incubated at 4 °C overnight. The proteins were collected by centrifugation at 1700 ***g*** for 30 min at 4 °C. The pellet was washed using 1/5th volume ice-cold acetone for 2 h at −20 °C, recentrifuged, the supernatant removed, and the pellet air dried for 10 min, followed by resuspension in 800 µl 50 mM sodium acetate pH 5.5. Protein quantification was performed using Bradford reagent (Biorad).

### PreNsoA1-4 leader cleavage assays

Protein expression from *E. coli* BL21 (DE3) strains, crude protein extraction and quantification and confirmation of expression by Western blotting were performed as described previously [53], with changes to the induction time and temperature to 3 h and 18 °C, respectively. Both cell-free lysates and cell wall fractions were collected after bead beating. Cell wall fractions of *E. coli* strains expressing the candidate proteases (10 µg) and TCA-precipitated supernatants containing pre-NsoA1-4 peptides (30 µg) were made to 13 µl using 100 mM Tris HCl pH 6 supplemented with 5 mM CaCl_2_ and left at 37 °C overnight, as recommended by Montalban-Lopez *et al.* [54]. TCA-precipitated supernatant from *L. lactis* pIL253 was used as a negative control and 15 µg ml^−1^ trypsin as a positive control of leader cleavage. Samples were analysed by SDS-PAGE and Western blot using a polyclonal antibody to the NsoA1-3 leader as described previously [15].

### PepI reporter assay

The peptidase I gene (*pepI*) was used in promoter–reporter assays as described previously [55]. The *nso* promoters preceding *pepI* were predicted using BPROM [56] from the nisin O BGC from the genome of *B. obeum* A2-162; for further sequence details, refer to Fig S2 and Table S2. *L. lactis* strains containing two plasmids expressing either the constitutively expressed nisin O regulatory systems or *pepI* preceded by each putative *nso* promoter were grown overnight using antibiotic selection. A 2% inoculum was used in GM17, induced with 10 ng µl^−1^ nisin A or pre-NsoA1-4 peptides (after overnight incubation with 15 µg ml^−1^ trypsin) and grown until the OD_600_ reached 0.5. The cultures were normalized to 10 OD units and harvested by centrifugation at 10 000 ***g*** for 15 min at 4 °C. The supernatant was discarded, and the pellets were washed with 50 mM Tris HCl (pH 7.4). The pellets were resuspended in 50 mM Tris HCl (pH 7.4) and bead beaten with acid-washed beads using a FastPrep-24 homogenizer (MP Biomedicals) for 4 × for 30 s at speed 6 with 5–10 min on ice between beating followed by centrifugation at 14, 000 ***g*** for 30 min at 4 °C. Dilutions of the crude cell-free extract were made in 50 mM Tris HCl (pH 7.4), and 50 µl of undiluted or diluted samples was incubated with 0.7 mM l-proline p-nitroanilide trifluoroacetate salt to measure PepI activity using a Benchmark Plus plate reader (Biorad) at A405 at 32 °C for 15 min. Protein quantification of the crude cell free extract was performed using Bradford reagent (Biorad). The rate of activity was calculated in nmol nitrophenol released/mg protein/min.

### cDNA synthesis and long-read sequencing

*L lactis* UKLc10 p*nso* was grown overnight at 30 °C in GM17 with erythromycin selection and nisin A induction (10 ng ml^−1^). Cells were harvested by centrifugation at 1700 ***g*** at 4 °C for 10 min, supernatant was discarded, and cells were lysed using 100 µl lysozyme (15 mg ml^−1^), 10 µl proteinase K (20 mg ml^−1^) and 3 µl mutanolysin (10 U µl^−1^) at room temperature for 10 min. RNA extraction was carried out using the RNeasy Mini Kit (Qiagen) following the manufacturer’s instructions with two rounds of on-column DNase treatment. The absence of genomic DNA was confirmed by conventional PCR of the 16S rRNA gene using primers Amp_F and Amp_R [57] (Table S1). RNA was quantified using a Qubit Broad Range RNA Quantification Kit (Thermo Fisher Scientific) and Qubit 3.0 fluorometer (Life Technologies). Before Poly-A addition, RNA was incubated at 70 °C for 2 min and cooled on ice. Polyadenylation was performed using the *E. coli* poly(A) polymerase (New England Biolabs), following the manufacturer’s instructions. The reaction was stopped by directly purifying the RNA with the Qiagen RNeasy Micro clean-up protocol. Poly(A) tailing was verified using a Bioanalyzer (Agilent), then rRNA was depleted using NEBNext rRNA Bacteria Depletion Kit using the manufacturer’s protocol, and the reaction was stopped and cleaned as before. Library construction for the direct ONT cDNA sequencing was performed using the manufacturer’s instructions (Oxford Nanopore Technologies, SQK-DCS109) on a PromethION Flow Cell (Oxford Nanopore Technologies). Raw reads were quality profiled with fastp v.0.20.0 [58] (RRID:SCR_016962) and filtered by length (>100 bp) using SeqFu v.1.16 [59]. Reads were mapped to the heterologous expression system genome and p*nso* plasmid using Minimap2 using the ‘map-ont’ preset [60] (RRID:SCR_018550). The mapping coverage from the output BAM file [61] was profiled using BamToCov v.2.7 [62] and plotted with pyGenomeTracks v.3.6 [63] (RRID:SCR_025312).

### Tertiary structure prediction of Nso1.14

The amino acid sequence of *B. obeum* A2-162 Nso1.14 was used as the input for AlphaFold2 analysis using MMseqs2 (v.1.5.5) [64].

## Results

### Nisin O-like BGCs are found in *Blautia*, *Ruminococcus* and *Dorea* species

**Fig. 1.**
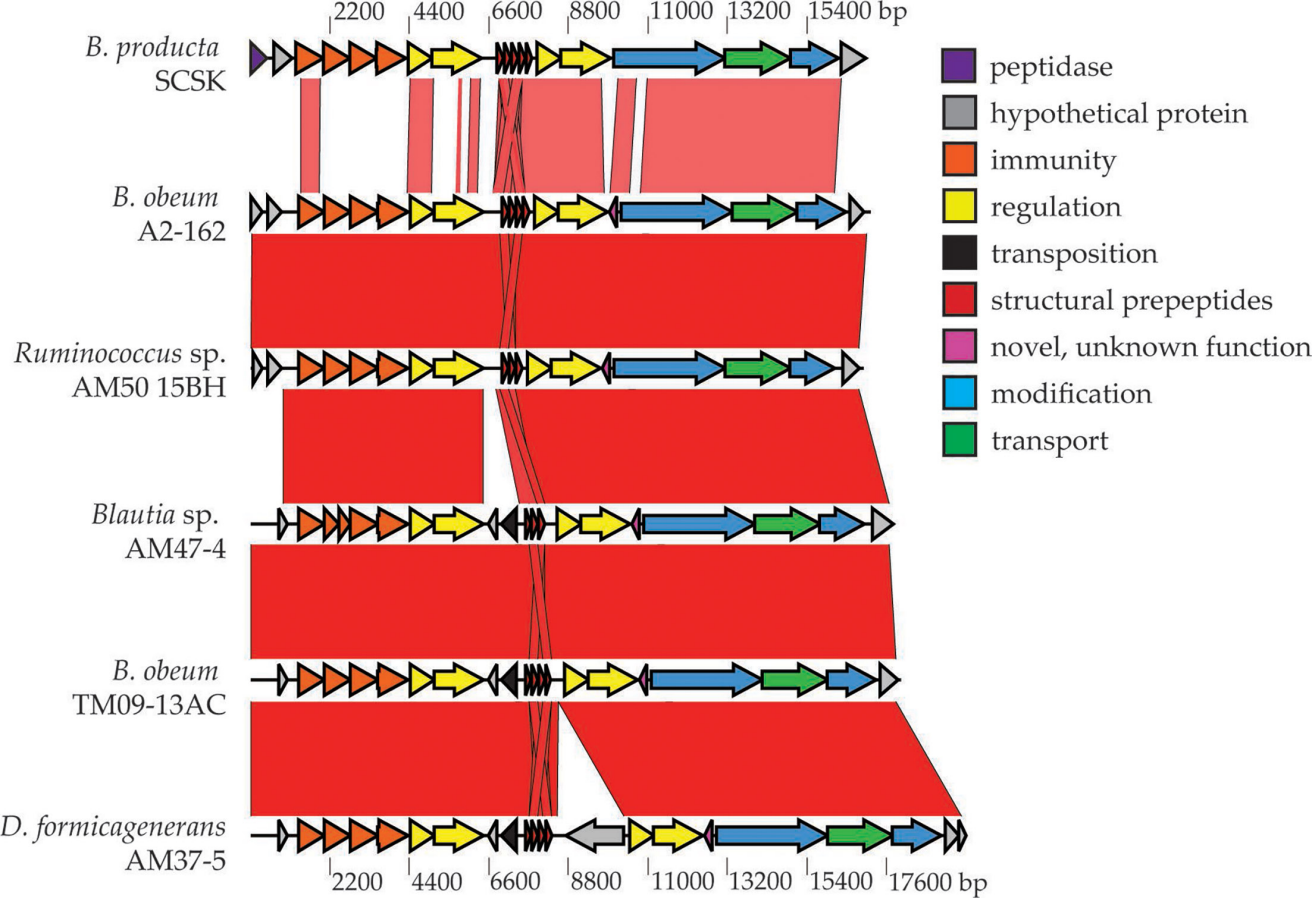
ACT cluster analysis of the nisin O-like BGCs and the *B. producta* SCSK cluster nucleotide sequences. The *D. longicatena* 1001136B-160425 BGC was omitted due to its high nucleotide similarity (99%) to the *Ruminococcus* AM50-15BH BGC. Purple, *lanP* (signal peptidase); orange, immunity; yellow, RK systems; red, structural prepeptides; pink, novel antisense gene of unknown function (*nso1.14*); blue, modification; green, transport; grey, hypothetical proteins; black, proteins with homology to transposases or transposase domains.

**Fig. 2.**
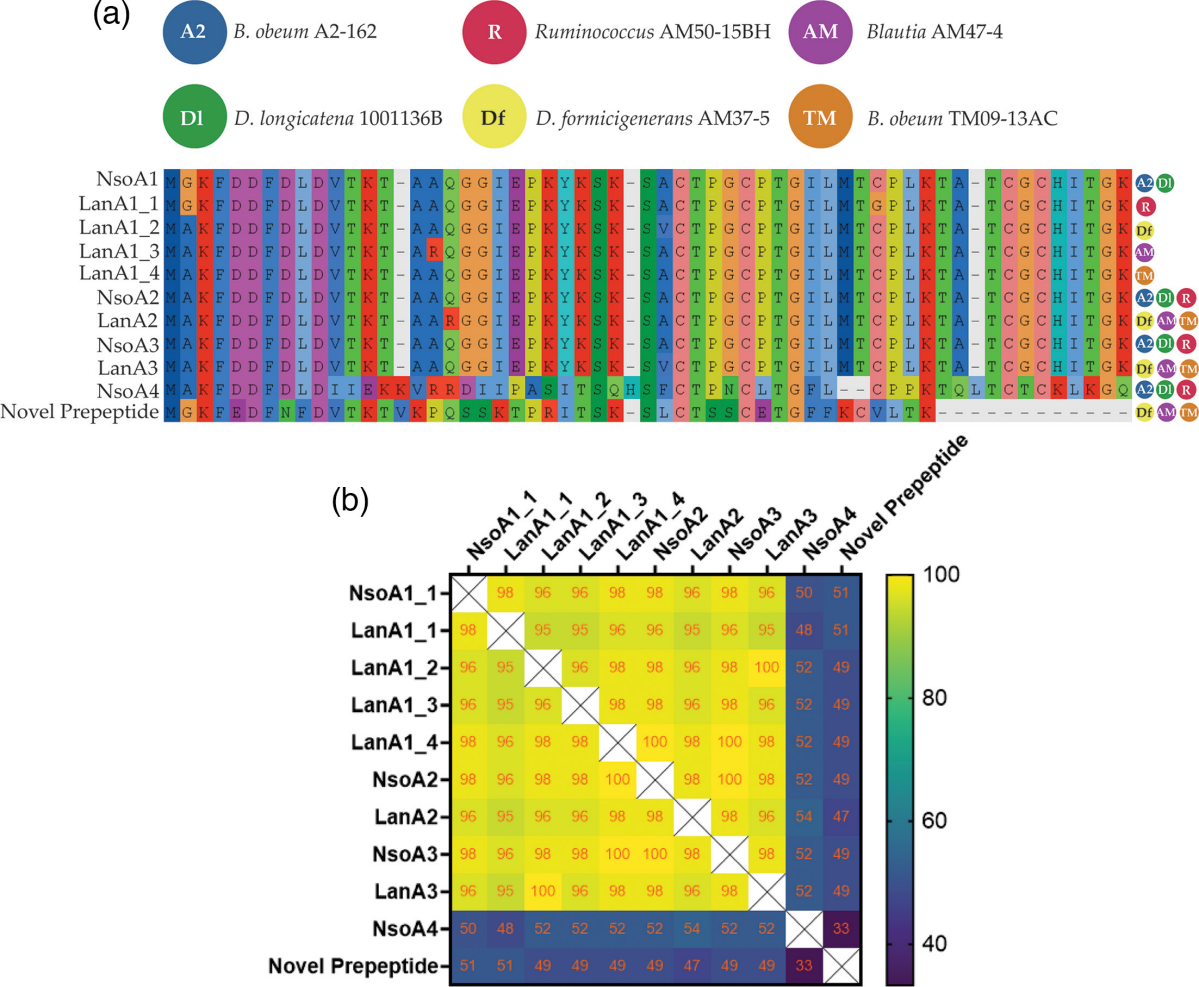
**(a)** Alignment of LanA pre-peptide amino acid sequences from novel nisin O-like clusters compared with NsoA1-4 from *B. obeum* A2-162. The identical novel prepeptide identified in *B. obeum* TM09-13AC, *Blautia* AM47-4 and *D. formicigenerans* AM37-5 is also included. Coloured dots (right of each sequence) indicate the bacteria in which the variant of the LanA peptide or novel prepeptide was identified. (**b) **Heat map representing the percentage amino acid identity of the 11 LanA prepeptide sequences identified in the nisin O-like clusters. Values within squares represent amino acid percentage identity.

In this study, we aimed to investigate the prevalence of the nisin O cluster across bacterial species, to understand its distribution and the conservation of novel features such as dual regulatory systems and to identify potential leader cleavage proteases which may have been lost from the *B. obeum* A2-162 nisin O cluster. An initial high stringency blastp search using the NsoB amino acid sequence to search for closely related clusters identified novel homologues in *B. obeum* AM27-32LB, *B. obeum* TM09-13AC, *Dorea longicatena* 1001136B-160425 and *Mediterraneibacter* (formerly *Ruminococcus* [65]) *gnavus* AF33-12 (only identified using BAGEL4), *Blautia* sp. AM47-4, *Ruminococcus* sp. AM50-15BH and *Dorea formicigenerans* AM37-5 [66]. A new study of blauticin-like LanAs also identified nisin-like peptides in the last three strains and demonstrated activity using a heterologous expression system [34]. Manual searches within these genomes along with antiSMASH and BAGEL4 analysis identified a full lantibiotic BGC in each of these bacterial strains. The bacteria identified were all members of the family *Lachnospiraceae*; therefore, a pan-genome gene family cluster analysis using OrthoFinder was performed to understand the distribution of the nisin O BGC within this family through the identification of the two key biosynthetic marker genes *nisB* and *nisC*. Two thousand ninety-four *Lachnospiraceae* genomes were used in this analysis, and after the removal of 664 genomes that did not pass the quality and completeness criteria, the remaining 1430 genomes were analysed using antiSMASH v.5.0 (bacterial version) for the presence of *nsoB* and/or *nsoC* genes as these were distinct for lantibiotic BGCs, whereas other genes had multiple, highly similar homologues that were not part of any clusters. The lantibiotic structural *nsoA1-4* genes were not used due to their small size. Orthofinder identified 59 genomes containing either *nsoB*, *nsoC* or both, and these were analysed using antiSMASH and BAGEL4 for the presence of antimicrobial BGCs (Table S3). Fifty-eight per cent of the genomes (34/59) contained both *nsoB* and *nsoC* genes with antiSMASH and BAGEL4 analysis identifying nisin O-like clusters in five out of the seven bacterial species detected with the blast approach: *B. obeum* AM27-32LB, *B. obeum* TM09-13AC, *Blautia* sp. AM47-4, *Ruminococcus* sp. AM50-15BH and *D. formicigenerans* AM37-5; however, *D. longicatena* 1001136B-160425 was not identified in this analysis, indicating the value of using both bioinformatic methods for BGC identification. The *B. obeum* AM27-32LB ORF had multiple frameshifts so may not be a functional cluster. Three additional novel lantibiotic clusters were identified in *Pseudobutyrivibrio* sp. UC1225, *Pseudobutyrivibrio* sp. 49 and *M. gnavus* AF33-12 [66], with the latter two clusters having their LanAs also identified recently by Zhang *et al.* [34]. However, these showed poor cluster synteny and low amino acid homology compared to the nisin O BGC (Fig S3-9). All clusters identified in *Blautia*, *Dorea* and *Ruminococcus* AM50-15BH have a high level of synteny with the nisin O BGC (Fig. 1), with the percentage nucleotide identity ranging from 98.4 to 100% across the whole cluster compared to the *B. obeum* A2-162 nisin O BGC (Fig. S3). The six nisin O-like BGCs had variable numbers of *lanA* genes, including homologues to *nsoA1-3* and *nsoA4*, and in three cases (*B. obeum* TM09-13AC, *Blautia* AM47-4 and *D. formicigenerans* AM37-5) a new prepeptide with homology to WP_116740231 (multispecies gallidermin/nisin sequence) (Fig. 2). These three also all had one additional prepeptide inactivated by frameshifts. LanA1-4 (Figs 2, S4–7), LanB and LanC amino acid sequences (Figs S8 and S9) showed high percentage identities between the clusters, with the greatest differences being with the LanA4 (32.1%) of *D. formicigenerans* AM37-5 and the LanB (56.7%) of *B. obeum* AM27-32LB (which had a large number of frameshifts within the BGC) when compared to corresponding genes in the *B. obeum* A2-162 BGC (Figs 2, S8 and S9). This demonstrates that close variants of the same nisin BGC are found within multiple genera.

Three of the clusters are interrupted by genes predicted to be associated with transposition before and/or after the structural prepeptides (Fig. 1), and *B. obeum* A2-162 nisin O has a potential transposase downstream of the cluster [15]. Furthermore, all clusters contained an IS66 family insertion sequence (WP_005343038.1) (97.5% nucleotide pairwise identity) 106 bp downstream of LanC (Fig. S10). *B. obeum* A2-162, *D. longicatena* 1001136B-160425 and *Ruminococcus* sp. AM50-15BH all contain a site-specific integrase (WP_006859226.1) (7.5 kb downstream). *D. formicigenerans* AM37-5, *Blautia* AM47-4 and *B. obeum* TM09-13AC all contain a conjugal transfer protein (WP_004613393.1) and a recombinase family protein (WP_004613394.1) (7.14 kb and 7.42 kb downstream, respectively). The region between the end of the cluster and these genes has a nucleotide pairwise identity of 94.6%. Upstream of the cluster, there are no predicted transposase genes; however, in *Ruminococcus* sp. AM50-15BH, there is an IS66 family insertion sequence (WP_005422931.1) (5.45 kb upstream), and in all genomes, there is an antisense cluster of a relaxase/mobilization nuclease domain-containing gene (WP_002594786.1) and *mobC* (WP_015543475.1) (1.74 kb upstream), which are both involved in horizontal gene transfer (HGT) of genetic elements from plasmids into the genome [67]. The similarity of the sequence up- and downstream of the BGCs extends ~36 kb (73.7% pairwise identity) and 9 kb (88.8% pairwise identity), respectively (Fig. S10). The end of the gene homology upstream is after a CD0415/CD1112 family protein (WP_015543459.1), with homology to the conjugal transfer protein TrbL. Gene homology ends downstream after a site-specific integrase/recombinase family protein mentioned previously (Fig. S10). This suggests a region of homology of ~57.9 kb surrounding and including the nisin O BGC. The presence of transposases and other genes involved in HGT within and surrounding the cluster may suggest the potential of the whole or part of the nisin O cluster to be capable of transposition across bacterial species.

**Fig. 3.**
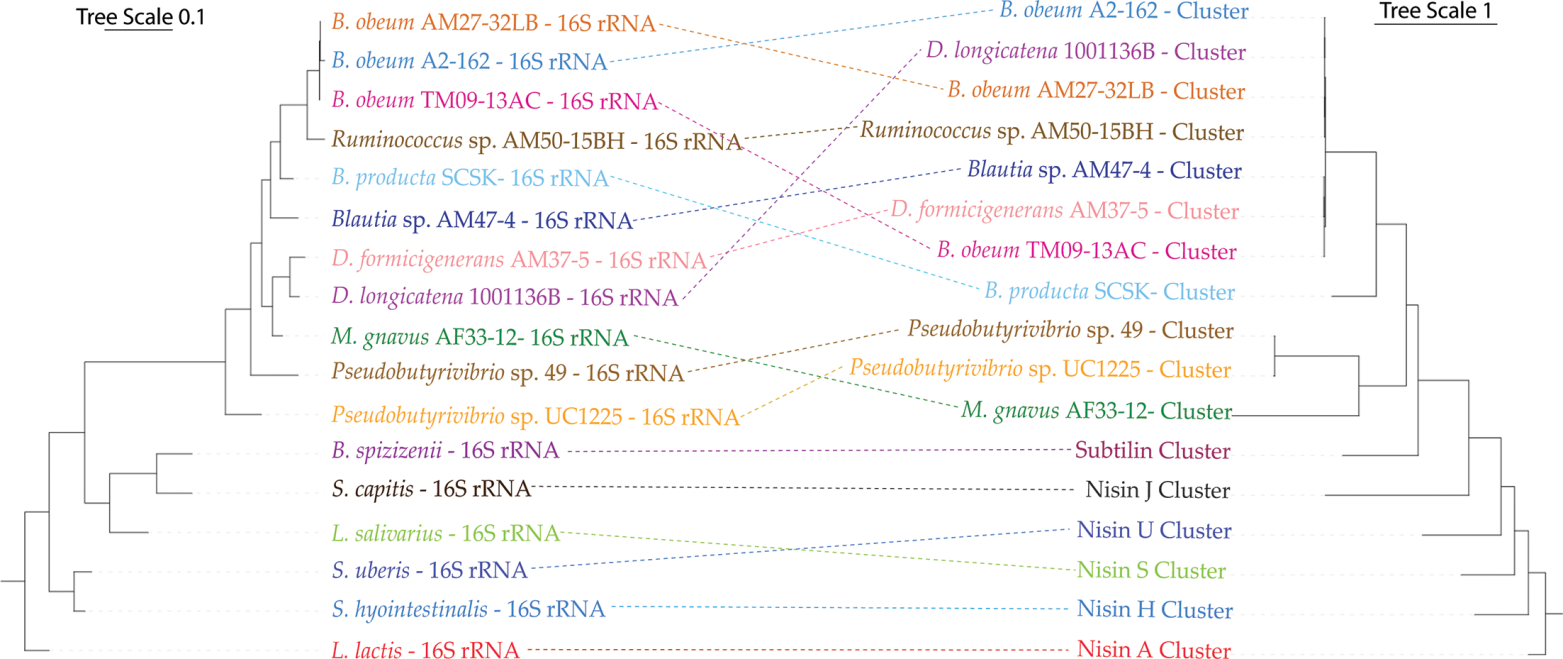
Maximum likelihood trees of the 16S rRNA sequence of each of the genomes in which a nisin O-like BGC was found (left) and whole BGC nucleotide sequences (right) identified through blastp and pan-genome analysis of *Lachnospiraceae*, including *B. producta* SCSK [31], nisins A (*L. lactis*) [87], U (*Streptococcus uberis*) [13], H (*Streptococcus hyointestinalis*) [14], S (*L. salivarius*) [19], J (*Staphylococcus capitis*) [17] and subtilin (*Bacillus spizizenii*) [81] clusters and 16S rRNA sequences of their respective bacterial species.

The generation of phylogenetic trees using the nucleotide sequence of the whole BGCs (Fig. 3, right) and the 16S rRNA sequence of the bacteria (Fig. 3, left) showed that the grouping of the nisin O-like BGCs clustered independent of the species/genera in which they were found. Conversely, the nisin A, U, S, J and H BGCs, in addition to the subtilin BGC, clustered in a similar branch pattern as their corresponding 16S rRNA genes, when *L. lactis* and the nisin A cluster were used to root the phylogeny. Together with the fact that the average GC percentage of the nisin O-like clusters (31.8%) differed clearly from the whole genomes (41.7%) (Fig. S11), this indicates that the nisin O BGC is potentially being disseminated by HGT within the human gut.

Of the 59 *Lachnospiraceae* genomes containing one or both of *nisB* and *nisC* homologues, only 12 did not contain a BGC containing structural bacteriocin genes; however, these genomes did contain individual genes involved in bacteriocin maturation. Interestingly, 42 of the 59 genomes contained lantibiotic immunity genes, even when no cluster was present (Table S3). It is known that the main habitat for *Lachnospiraceae* is the gut [68]; we found that 56 of the genomes were of human or animal origin, and the remaining 3 genomes were of bacteria isolated from other environments. All bacteria that contained the nisin O-like BGCs were isolated from the gut environment. It is possible that genetic transfer of individual elements of lantibiotic BGCs, such as immunity genes, occurs across taxa within the human gut, or there could be selective loss of unnecessary cluster components.

All nisin O-like BGCs lacked a protease within or near their clusters. Although there is some synteny with the *B. producta* SCSK cluster, it is lost in the region where the *lanP* signal peptidase is located upstream of the immunity genes (Fig. 1).

### Identification and functional analysis of NsoA1-4 leader peptide-cleaving candidates

As no nisin O BGC identified in the genome mining results contained a protease and the cleavage of the leader peptide is integral to antimicrobial activity, the *B. obeum* A2-162 genome was interrogated for candidate proteases based on the amino acid sequence of LanP (*B. producta* SCSK) using tblastn, conserved domains of LanP and manual searches for subtilisin-like proteases. We identified six proteases (Table S4) sharing between 12 and 18% amino acid identity to NisP through manual searches (Fig. S12). We selected proteins P66 (CBL24066, predicted subtilisin-like serine protease which had similar conserved domains and 18% amino acid homology to NisP) and P570 (CBL2470, a predicted trypsin-like serine protease with 14.6% homology to NisP) (Fig. S12). Three predicted signal peptidases (Table S5) were found with homology to *B. producta* SCSK LanP after tblastn and conserved domain searches. All these peptidases were successfully cloned; however, only P140 (CBL24140, 23.8% homology, Fig. S12), which shared the conserved domain 26_SPase_1 (PF10502), was shown to be expressed.

**Fig. 4.**
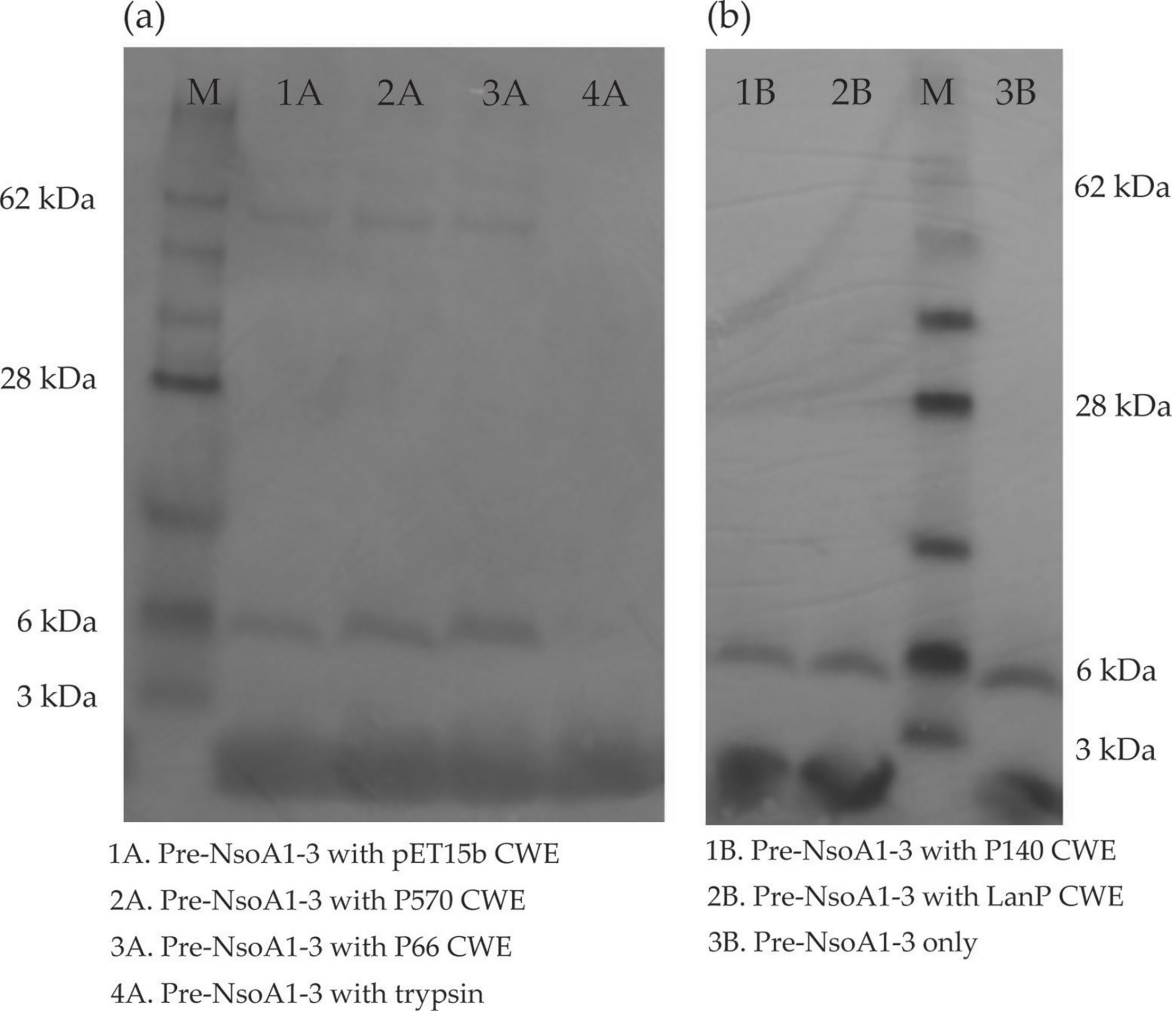
Pre-NsoA1-3 cleavage assays using cell wall extracts (CWE) from protease-expressing strains. (**a) **P570 and P66 and (b) P140 and LanP or pET15b controls that were incubated with TCA-precipitated supernatants from *L. lactis* UKLc10 p*nso* (Pre-NsoA1-3) with the presence/loss of the leader peptide detected on Western blots using a NsoA1-3 leader antibody. The marker (M) used was SeeBlue™ Plus2 Standard (Thermo Fisher Scientific). Pre-NsoA1-3 incubated with 15 µg ml^−1^ trypsin was used as a positive control.

To assess the potential leader peptide cleaving activity of candidate proteases, P570, P66, P140 and LanP were expressed as His-tagged proteins in *E. coli.* Nisin O cleavage assays were performed using either cell wall extracts of these expression strains or trypsin, incubated with TCA-precipitated supernatant of *L. lactis* UKLc10 p*nso* followed by a Western blot. An antibody to the leader peptide of NsoA1-3 (5.8 kDa) was used to assess whether the leader peptide (2.65 kDa) had been cleaved from the core peptide, resulting in the loss of the 5.8 kDa prepeptide band. After incubation with trypsin, there was no hybridization of the leader antibody at 5.8 kDa, indicating cleavage of the leader (Fig. 4a). However, none of the expressed candidate proteases caused loss or visible reduction of the prepeptide band, indicating that they were unable to perform cleavage of the leader peptide of NsoA1-3 (Fig. 4a, b). The smaller band (2.65 kDa) of the leader peptide produced by the cleavage by trypsin is not clearly visible, due to the loading dye-front.

### Interaction of nisin O regulatory systems with upstream regions of predicted operons

**Fig. 5.**
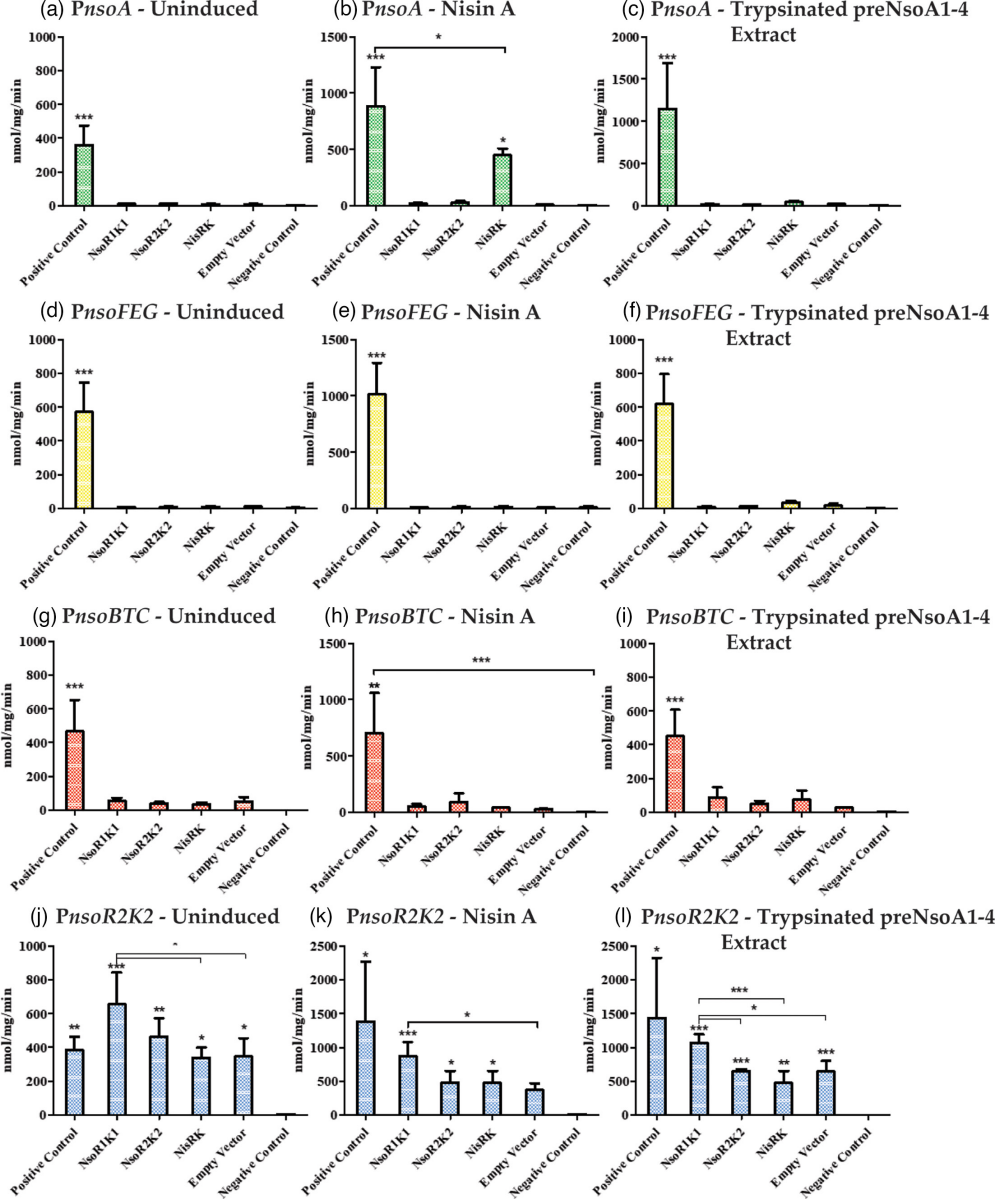
Promoter reporter assays showing rates of activity of PepI controlled by predicted nisin O promoters in combination with RK systems. Regulatory systems pUK200_P32_*nsoR1K1*, pUK200_P32_*nsoR2K2*, chromosomally expressed *nisRK* or empty vector control pUK200_P32 were co-expressed with promoter reporter constructs p*PnsoA*_*pepI* (**a–c**), p*PnsoFEG*_*pepI* (d**–f**), p*PnsoBTC*_*pepI* (**g–i**) and p*PnsoR2K2*_*pepI* (**j–l**) with no induction (**a, d, g, j**), induced with nisin A (**b, e, h, k**) or induced with trypsinated pre-NsoA1-4 precipitated from *L. lactis* UKLc10 p*nso* supernatant (**c, f, i, l**). Positive control, *L. lactis* UKLc10 strain with *nisRK* on the chromosome with p*PnisA_pepI*; negative control, *L. lactis* MG1614 pIL253. Significance was determined by a one-way ANOVA followed by a Tukey multiple comparison test. *P*-values of <0.05, <0.01 and <0.001 are indicated by ‘*’, ‘**’ and ‘***’, respectively. The asterisks directly above each column indicate the significance compared to the negative control unless indicated otherwise. Results are the mean of triplicate readings per induction condition from three separate experiments with error bars representing the standard deviation of the mean.

The pan-genome analysis showed that the six nisin O-like BGCs identified all contained two RK regulatory systems, unlike most nisin variant clusters. Nisin A biosynthesis is autoregulated by the NisRK system, which interacts with nisin to induce the expression of *nisABTCIPRK* and *nisFEG* [9]. We investigated interactions between the two regulatory systems and putative nisin O cluster promoters to understand how this cluster is regulated. This would provide further information on how this system could be induced and how the regulation of the nisin O cluster differs from other nisin variants. We performed promoter reporter assays in a two-plasmid system combining constitutively expressed nisin O RK systems (*nsoR1K1* and *nsoR2K2*) with the four predicted promoters (P*nsoFEGIR1K1*, P*nsoA*, P*nsoR2K2* and P*nsoBTC* [69]) fused to reporter gene *pepI* in *L. lactis* MG1614. As previous work indicated improved prepeptide production with nisin A induction [15], the nisin A RK system was chromosomally expressed in *L. lactis* UKLc10 with the predicted promoter-reporter gene system expressed on plasmids to investigate cross-induction. Promoter activity was measured with and without induction with nisin A or trypsinated pre-NsoA1-4 extract. The P*nsoA* promoter showed no activity when uninduced or in the presence of trypsinated pre-NsoA1-4 extract (Fig. 5a, c). However, when induced with nisin A, there was an increased level of activity when the nisin A *nisRK* system was used (Fig. 5b). There was no notable activity under any induction condition for P*nsoFEG* (Fig. 5d-f). The P*nsoBTC*-containing strain showed a very low level of activity for all induction conditions, including the empty vector control, which indicates that this promoter has a low level of expression. However, the expression seen in the presence of the regulatory systems was not significantly different from those of the empty vector control, indicating that neither NsoR1K1 nor NsoR2K2 interacted with the P*nsoBTC* promoter (Fig. 5g-i). The P*nsoR2K2* promoter showed the greatest levels of activity across all induction conditions (Fig. 5j-l). The activity was seen in the empty vector control, indicating that this promoter is constitutive. When the NsoR1K1 system was also present, there was a significant increase in activity when compared to the empty vector control and the NisRK system in all induction conditions. This suggests that there is interaction between the NsoR1K1 system and the P*nsoR2K2* promoter to increase expression.

### Long-read RNA sequencing indicates high expression levels of *nsoA1-4* and *nsoR2K2* and a newly identified anti-sense gene, *nso1.14*

**Fig. 6.**
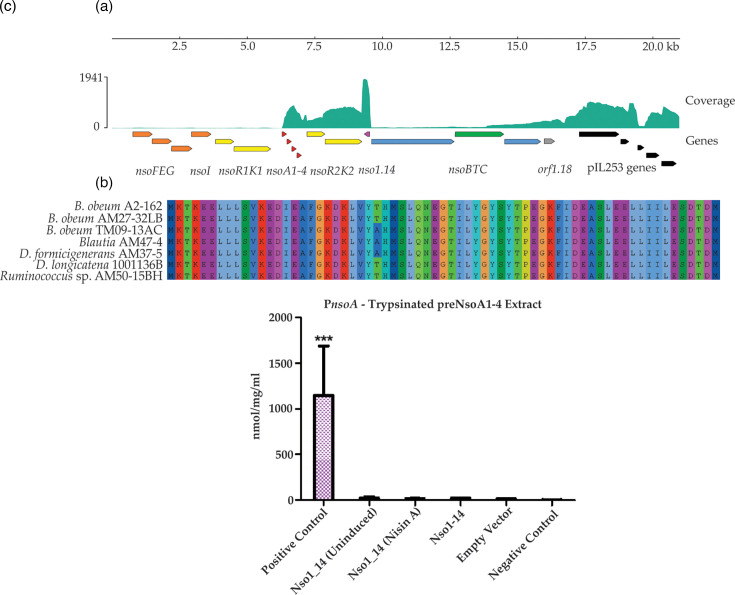
**(a) **Mapped reads of cDNA against the p*nso* sequence containing the nisin O BGC. Long-strand RNA was extracted from nisin A-induced *L. lactis* UKLc10 p*nso* and used for Nanopore sequencing. Orange, immunity; yellow, RK systems; red, structural prepeptides; pink, novel antisense gene of unknown function (*nso1.14*); blue, modification; green, transport; grey, hypothetical protein; black, expression plasmid genes.(**b)**Alignment of Nso1.14 homologues from the nisin O-like BGCs. (**c) **The rates of activity of PepI expressed by pUK200_P32_*nso1.14* and pP*nsoA*_*pepI* containing strains when induced with trypsinated pre-NsoA1-4, nisin A or uninduced. The empty vector control (pP*nsoA*_*pepI* pUK200_P32), positive control (*L. lactis* UKLc10 pP*nisA*_*pepI*) and negative control (*L. lactis* MG1614 pIL253) were all induced with trypsinated pre-NsoA1-4. Significance was determined by a one-way ANOVA followed by a Tukey multiple comparison test. *P*-values of <0.05, <0.01 and <0.001 are indicated by ‘*’, ‘**’ and ‘***’, respectively. The asterisks directly above each column indicate the significance compared to all other strains unless indicated otherwise. Error bars were made using the standard deviation of the mean. Each induction condition was repeated in triplicate.

To understand which genes were being expressed in the heterologous expression system, Oxford Nanopore Technology long-read sequencing was performed on the cDNA of nisin A-induced *L. lactis* UKLc10 p*nso*. This produced 2.4 million reads (min 29, max 239012, with 16.7% of reads >1 kbp). When the reads were mapped to the nisin O BGC (0.27%), it was found that there were high levels of expression of *nsoA1-4* and *nsoR2K2* and a previously unidentified gene, transcribed in the antisense direction, *nso1.14* (Fig. 6a). There were very low levels of transcription of *nsoFEG*, *nsoI* and *nsoR1K1*, which corroborate results seen in the PepI reporter assay. The four structural peptides were co-transcribed, indicating that all four peptides are under the control of the same promoter. Although modification enzymes *nsoBTC* are all transcribed, it appears that *nsoB* may be independently regulated, or there may be some 5′ instability of the transcript. There was increased read coverage at the end of the cluster covering *nsoC* and the downstream *orf1.18* before the highly expressed genes of the p*nso* cloning vector (Fig. 6a).

Comparative alignment analysis showed that the *nso1.14* gene was identified in all six nisin O-like BGCs and is highly conserved (Fig. 6b). The analysis using BPROM identified a predicted promoter in *B. obeum* A2-162 which is present in the antisense direction with the putative promoter region 20 nucleotide upstream of the start site of *nso1.14*. However, *nso1.14* was not present in the nisin A, U, H and Q BGCs or the *B. producta* SCSK BGC. The AlphaFold2 model of the *B. obeum* A2-162 Nso1.14 has very low confidence (maximum of 70 plDDT) (Fig. S13A, B), and further FoldSeek analysis did not elucidate any potential homologues to suggest a known function for this protein. Together with the observed high expression, this indicates that this gene may serve an important function within the BGC. To investigate this further, we performed a promoter reporter assay investigating the effect of constitutively expressed Nso1.14 on the predicted promoter of the other highly expressed transcript, pP*nsoA*, in *L. lactis*. PepI expression was measured using uninduced, nisin A-induced and trypsinated pre-NsoA1-4-induced conditions. None of these conditions induced promoter activity, suggesting that Nso1.14 is not directly involved in the induction of P*nsoA* expression (Fig. 6c).

## Discussion

Nisin and its variants have the potential to be developed as therapies to address the spread of antimicrobial resistance [31], and variants produced by gut microbes which may have evolved to function in the challenging environment of the gastrointestinal tract are particularly desirable. The nisin regulatory system has also been exploited as a valuable tool for controlled gene expression [9]. Further investigation of the regulation of new nisin variants may support the development of new antimicrobials. Nisin O has previously been shown to have antimicrobial activity against *C. perfringens* and *Clostridium difficile* [15], while recent work has demonstrated efficacy against vancomycin-resistant *Enterococcus faecium* (VRE), methicillin-resistant *Staphylococcus aureus* USA300 and methicillin-resistant *Staphylococcus epidermidis* SK135 [34], making it a potential candidate for antimicrobial therapy. However, due to the unusual structure of the cluster and the absence of some fundamental genes, it is necessary to understand in detail how this BGC functions to maximize production efficiency from the producer organism. In this investigation, we searched for closely related cultures and identified six full nisin O-like clusters in *Blautia*, *Ruminococcus* and *Dorea* species, in addition to three lantibiotic BGCs in *Pseudobutyrivibrio* and *Mediterraneibacter* species. We suggest the dissemination of this cluster or its parts between different genera of *Lachnospiraceae* is due to HGT [70]. A recent study found that *nisFEG* and *nisI* genes were present in 9% of *L. lactis* subsp. *lactis*, *L. lactis* subsp. *cremoris* and *L. lactis* subsp. *cremoris* with *L. lactis* subsp. *lactis* phenotype without other elements of the nisin cluster, and these strains were able to grow in high nisin concentration environments [71]. This suggests that immunity genes may provide a competitive advantage to the bacteria without having to bear the fitness cost and energy expenditure of lantibiotic biosynthesis.

Multiple genes predicted to be involved in transposition were identified within and surrounding the nisin O-like BGCs. All clusters contained an IS66 family insertion sequence 106 bp downstream and *mobC*/relaxase genes which are conserved across all BGC sequences and involved in HGT in the microbiota [72]. Previously, the nisin A cluster has been identified on the chromosomally located transposon Tn5301 (70 kb in size) [73] with subsequent work on a nisin-sucrose transposon observing the insertion of the nisin cluster into a nisin A-absent *L. lactis* strain [74]. This demonstrates that large transposable elements that contain nisin BGCs are capable of HGT, and therefore, this could be occurring in the human gut for niche clearing and/or bacterial community maintenance, thus providing a survival advantage. O’Connor *et al.* also demonstrated close relationships and conservation of gene order between other nisin clusters which may be a result of HGT [14]. Although genes with a close relationship to nisin O were restricted to the *Lachnospiraceae*, a wide distribution of immunity genes and a mechanism for them to be transferred to related species in the gut environment may be of concern for the development of nisin O as an antimicrobial.

In all nisin O-like clusters, no leader-cleaving peptidase was identified, although a related BGC from *B. producta* SCSK has a cluster-associated signal peptidase. Leader peptide cleavage is required for antimicrobial activity to occur, but the lack of a protease within the nisin O BGC is not unprecedented [19]. The absence of a protease is also seen in the ruminococcin A BGC, which requires trypsin both to induce expression and to cleave the leader peptide [75,76]. Like *B. obeum* A2-162, the producer of ruminococcin A, *M. gnavus* E1, is a member of the *Lachnospiraceae* family and was isolated from the human gut. Other lantibiotic systems cleave leader peptides using proteases outside of the BGC, such as the AprE, WprA and Vpr proteins which can cleave the leader peptide of subtilin [33]. Through genomic analysis of the *B. obeum* A2-162 genome, a number of candidate leader peptide cleavers were identified, followed by functionality testing. However, no leader peptide cleavage activity was observed using any candidate protease when expressed in *E. coli* in the pre-NsoA1-4 cleavage assay; leader peptide cleavage was only observed when trypsin was present. Trypsin has a high concentration in the intestinal tract (143.0 µg ml^−1^) and cleaves after a lysine or arginine residue [77]. NsoA1-3 prepeptides contain six trypsin cleavage sites, two of which are at the end of the leader peptide so trypsin can generate active NsoA1-3; however, there are also two sites within the core peptide which can lead to degradation of the peptide [78]. In a recent study investigating the lanthipeptide, *sapT,* in an *E. coli* expression system, the authors suggested that the lanthionine rings are able to provide protection of the core peptide from proteolytic degradation [79]. Thus, the lanthionine ring-containing core peptide of NsoA1-3 may have a greater protection from cleavage via trypsin compared to the leader peptide, allowing the nisin O BGC to use trypsin from the gut environment to generate active antimicrobials. However, the presence of a leader-cleaving protease in the genome is not ruled out. A candidate for the cleavage of the NsoA4 prepeptide, which does not have a trypsin site capable of removing the leader, remains unidentified, and both the function of this peptide and whether it is proteolytically processed merit further investigation.

The nisin O-like BGCs all contained two RK regulatory systems, which is again unusual for nisin variant BGCs. We used a PepI reporter assay to investigate the interactions between the two RK regulatory systems and the putative *nso* promoters. Neither NsoR1K1 nor NsoR2K2 induced expression from the predicted promoters P*nsoA*, P*nsoFEGIR1K1* and P*nsoBTC* in a *L. lactis* heterologous expression system. However, the P*nsoR2K2* promoter, which showed constitutive expression in this system, gave an increased level of activity when interacting with NsoR1K1. This suggests that NsoR1K1 may contribute to nisin O gene expression via up-regulation of NsoR2K2, but that NsoR2K2 either does not induce nisin O promoters or requires some external factor/s, genes or conditions absent from the heterologous system which would be present in the native *B. obeum* A2-162, or that the upstream regions we selected were not authentic promoters. The fact that an interaction between the NisRK system of nisin A and the P*nsoA* promoter was seen when nisin was present indicates this region is a true promoter and demonstrates a level of promiscuity between the regulatory systems of nisin variants. This has been seen before where nisin A, H and P were used to induce a green fluorescent protein reporter system with various levels of sensitivity [16] and represents a potential mechanism for optimising nisin O peptide production.

The data from the PepI reporter assay were supported in part by transcriptional analysis of long-read RNA sequencing. This confirmed high levels of expression of *nsoR2K2* and low expression of *nsoFEGIR1K1*. High constitutive expression of regulatory genes is also observed in the nisin A BGC [80]. The data also showed relatively high expression of *nsoA1-4* and medium expression of *nsoBTC* and suggest that the gene downstream of *nsoC* may also be expressed at the same time as the rest of the cluster. If this gene, which is highly conserved between clusters, is indeed related to transposition, it might be involved in the horizontal spread of this cluster between genera. Interestingly, transcriptional analysis also highlighted a previously unidentified highly expressed novel gene, *nso1.14*, encoded in the antisense direction, which was subsequently found in all six nisin O-like clusters. Constitutively expressed Nso1.14 did not induce expression from P*nsoA* in a heterologous system, but its transcription pattern suggests a role in the BGC. Complex layers of lantibiotic regulation have been observed previously in the regulation of subtilin which is controlled by SpaRK, whose expression in turn relies on sigma factor H [81]. Future work using a combination of reporter assays and transcription analysis in the native strain may help to resolve the factors involved in nisin O regulation.

In conclusion, we identified full nisin O-like clusters in multiple genera within the *Lachnospiraceae* family. Our analyses indicate that this BGC may have spread within this family via HGT. In highly competitive environments, such as the human gut, the acquisition of this BGC might facilitate colonization by preventing growth of competing species [82,83]. Our results confirm that trypsin is capable of leader peptide cleavage which could suggest why there is a lack of a candidate protease in all nisin O-like BGCs due to the energy cost of producing a protein which performs the same function as a secreted host protein. Furthermore, the newly identified gene in the cluster, *nso1.14*, is highly expressed, but its function has yet to be determined and could provide insight on the regulation of the cluster. Future work on this cluster could be focussed on a greater understanding of the two regulatory systems and identifying which environmental factors can influence the expression of nisin O.

## supplementary material

10.1099/mic.0.001531 Uncited Supplementary Material 1.

## Abbreviations

ACT: Artemis comparison tool
BGC: biosynthetic gene cluster
GTDB: Genome Taxonomy Database
HGT: horizontal gene transfer
NCBI: National Center for Biotechnology Information
RK: response regulator–histidine kinase
VRE: vancomycin-resistant *Enterococcus faecium*
