# Supplementary material for: The nisin O cluster: species dissemination, candidate leader peptide proteases and the role of regulatory systems
Source: Microbiology (Reading). 2025 Feb 10;171(2):001531. doi: 10.1099/mic.0.001531 (PMC11811420; doi:10.1099/mic.0.001531)
Supplement: Uncited Supplementary Material 1. [file mic-171-01531-s001.pdf]

The Nisin O Cluster: Species Dissemination, Candidate Leader Peptide Proteases and the Role of Regulatory Systems

Jacob Scadden[1•], Rebecca Ansorge[1,2‡], Stefano Romano[1◊], Andrea Telatin[1], Dave J. Baker[1], Rhiannon Evans[1†], Cristina Gherghisan-Filip[1], Zhenrun J. Zhang[3,4], Melinda J. Mayer[1*], Arjan Narbad[1]

[1]Quadram Institute Bioscience, Norwich Research Park, Norwich, UK

[2]Earlham Institute, Norwich Research Park, Norwich, UK

[3]Duchossois Family Institute, University of Chicago, Chicago, Illinois, USA

[4]Department of Microbiology, University of Chicago, Chicago, Illinois, USA

Current Addresses:

[•]School of Biotechnology and Biomolecular Sciences, University of New South Wales, Sydney, Australia

[‡]Department for Biological Safety, Federal Institute for Risk Assessment, Berlin, Germany

[◊]Structural and Computational Biology Unit, European Molecular Biology Laboratory, Heidelberg, Germany

[†]Department of Medical Genetics, University of Cambridge, Cambridge, UK

*Correspondence: Melinda J. Mayer, melinda.mayer@quadram.ac.uk

Supplementary Material - Figures S1-S13 and Tables S1-5

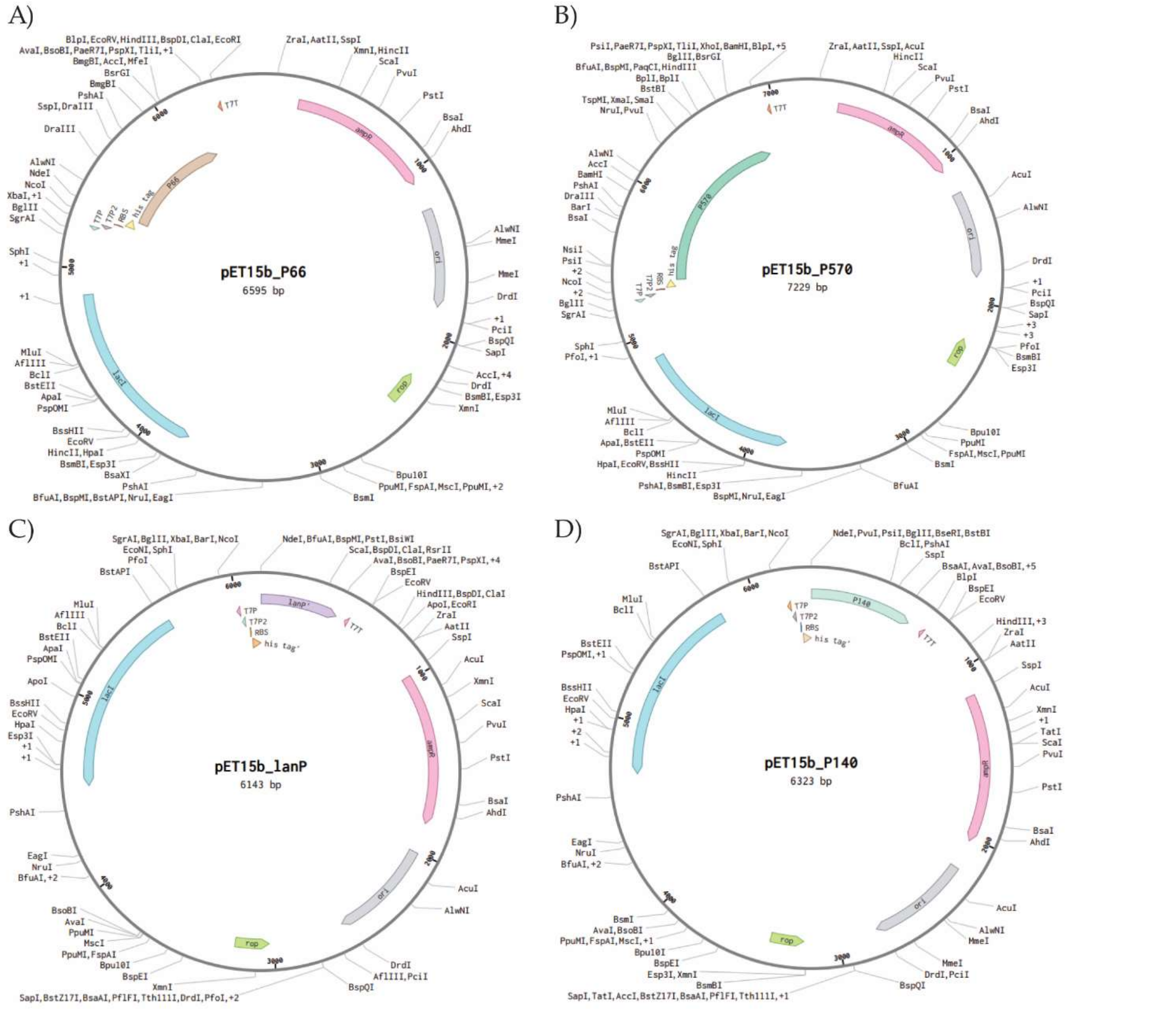

Figure S1. Plasmid maps of pET15b-derived plasmids used for candidate protease expression. Plasmids with candidate proteases *P66*, *P570*, *lanP* and *P140* are represented in panels A, B, C and D, respectively.

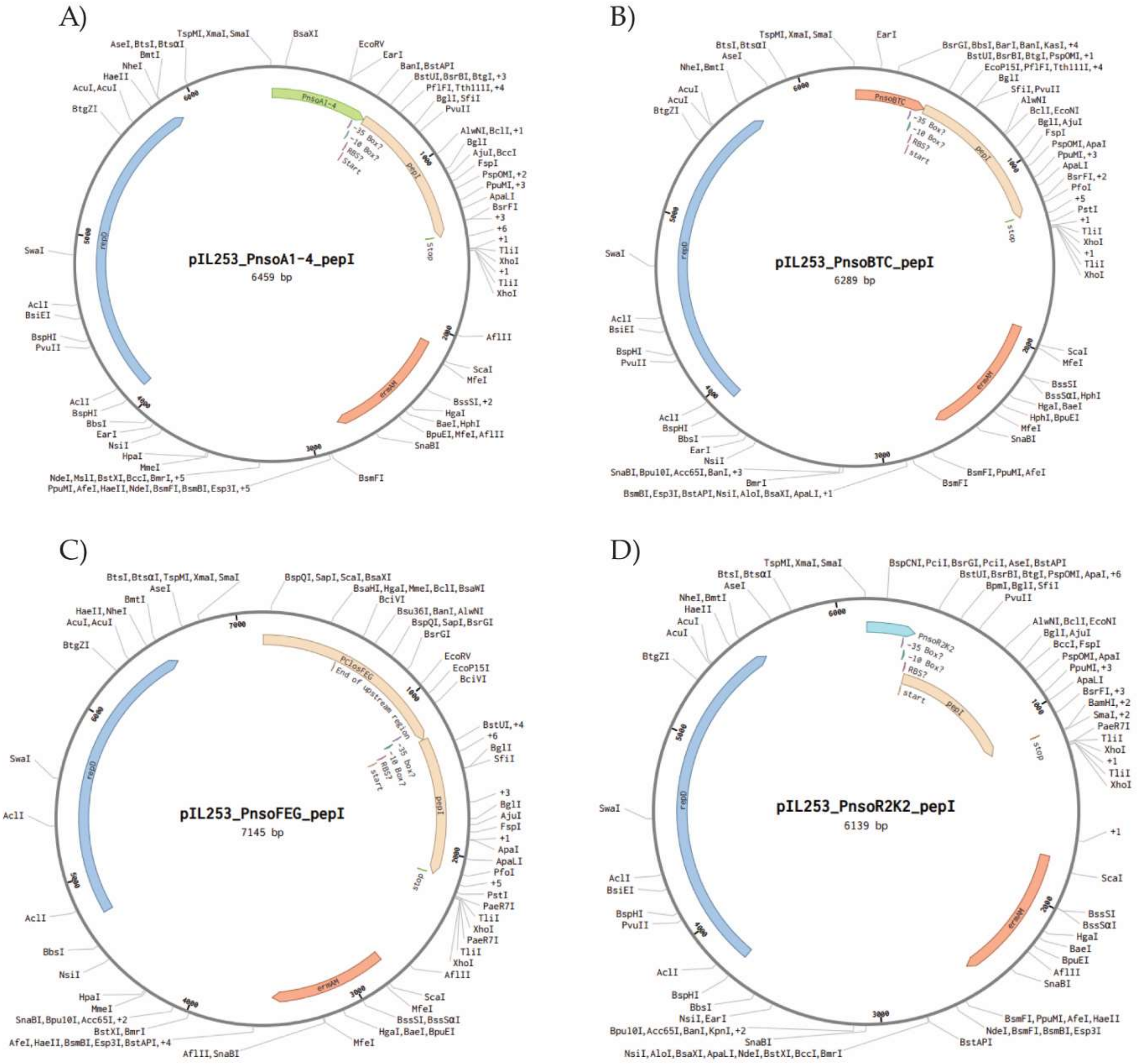

Figure S2. Plasmid maps of pIL253 derived plasmids used in *pepI* reporter assays. Plasmids with predicted promoter regions of *nsoA1-4*, *nsoBTC*, *nsoFEG* and *nsoR2K2* are represented in panels A, B, C and D, respectively.

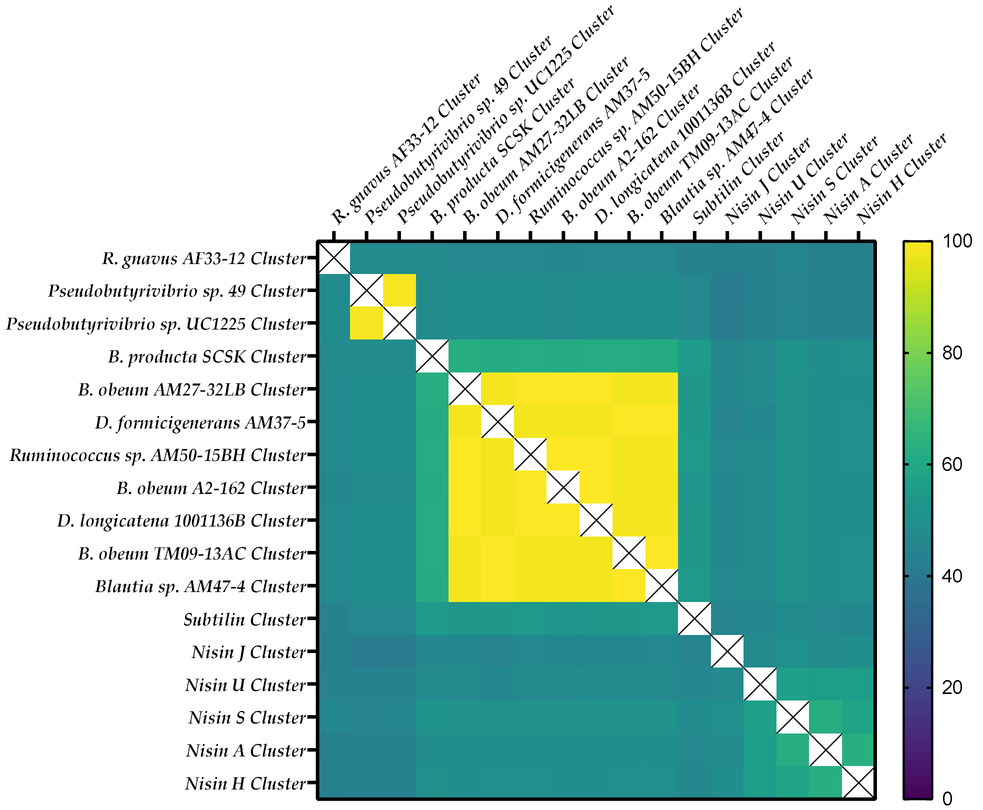

Figure S3. Percentage nucleotide identity heatmap of the nisin O, nisin O-like and previously identified nisin clusters.

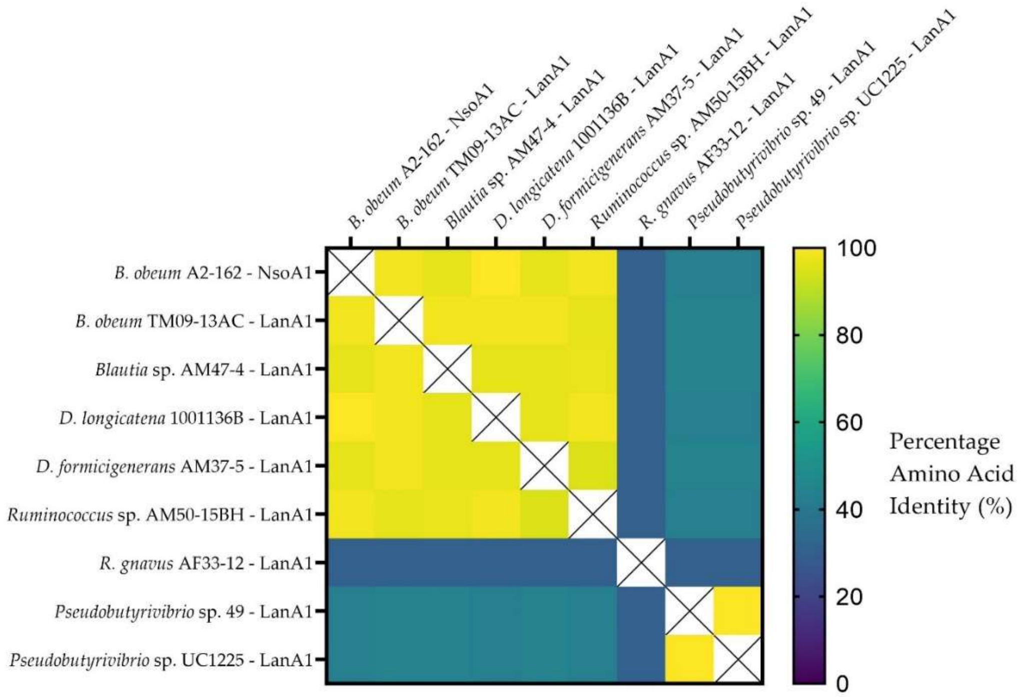

Figure S4. Percentage amino acid identity heatmap of NsoA1 and LanA1 from nisin O and nisin O-like clusters.

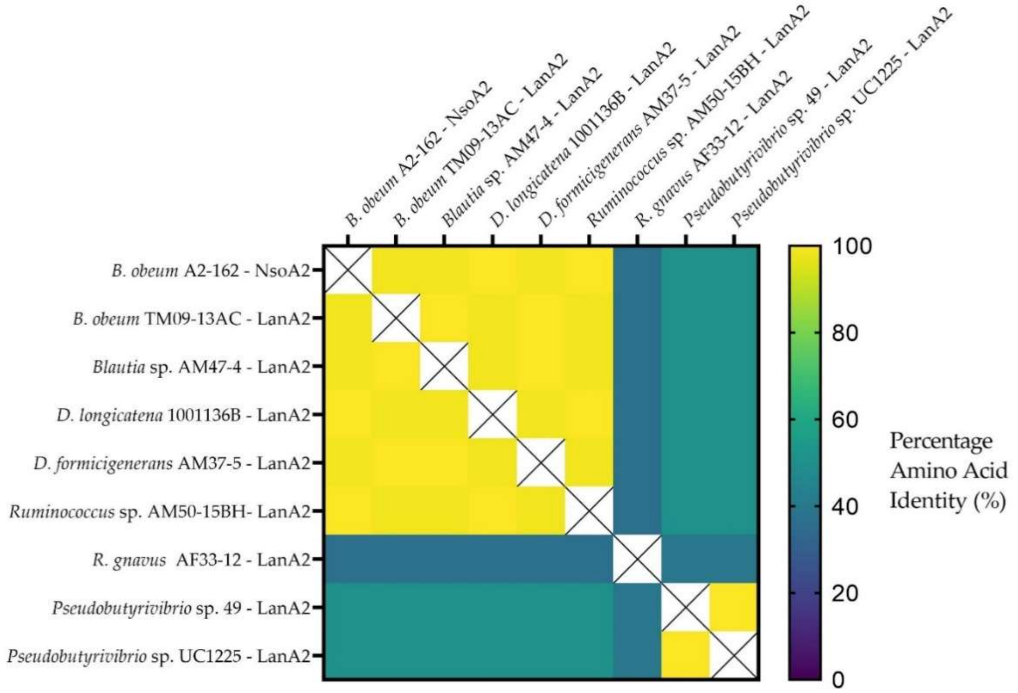

Figure S5. Percentage amino acid identity heatmap of NsoA2 and LanA2 from nisin O and nisin O-like clusters.

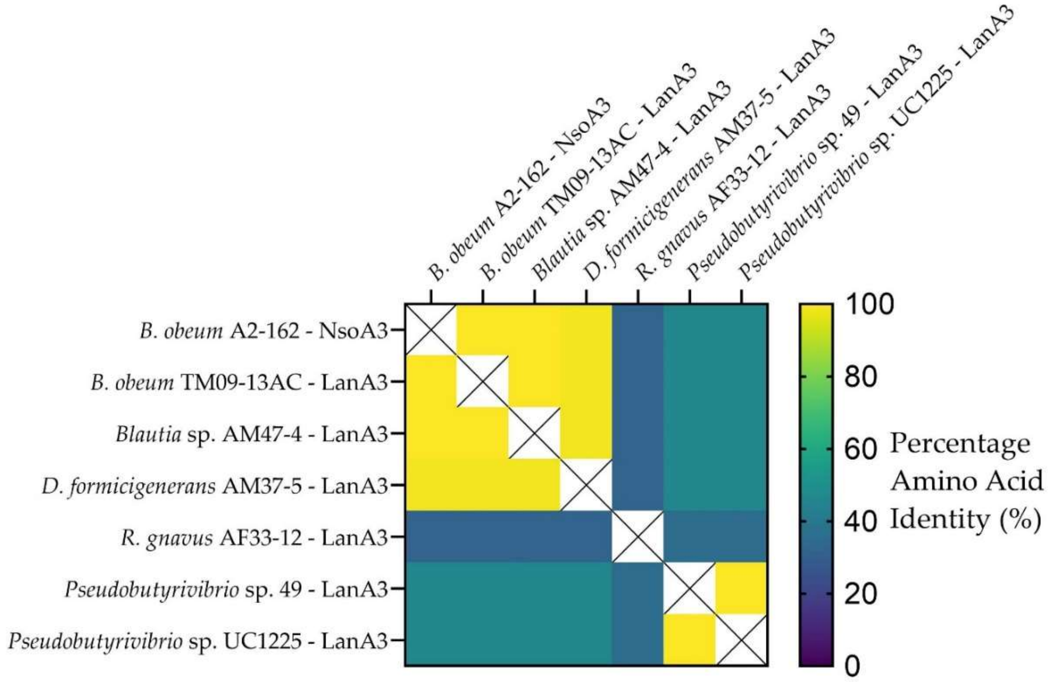

Figure S6. Percentage amino acid identity heatmap of NsoA3 and LanA3 from nisin O and nisin O-like clusters.

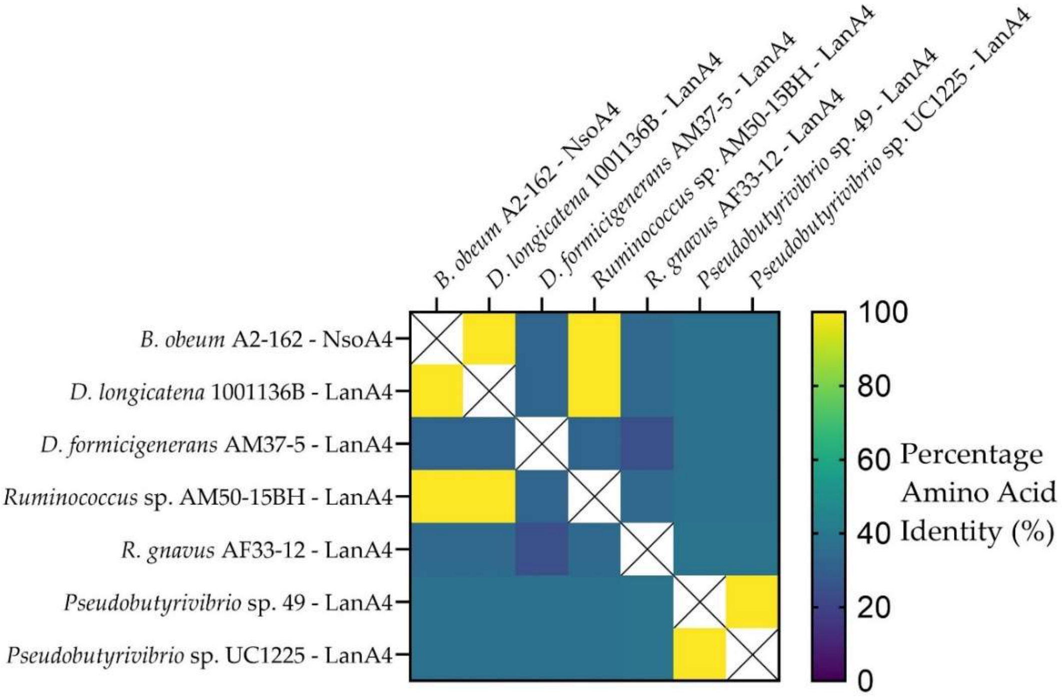

Figure S7. Percentage amino acid identity heatmap of NsoA4 and LanA4 from nisin O and nisin O-like clusters.

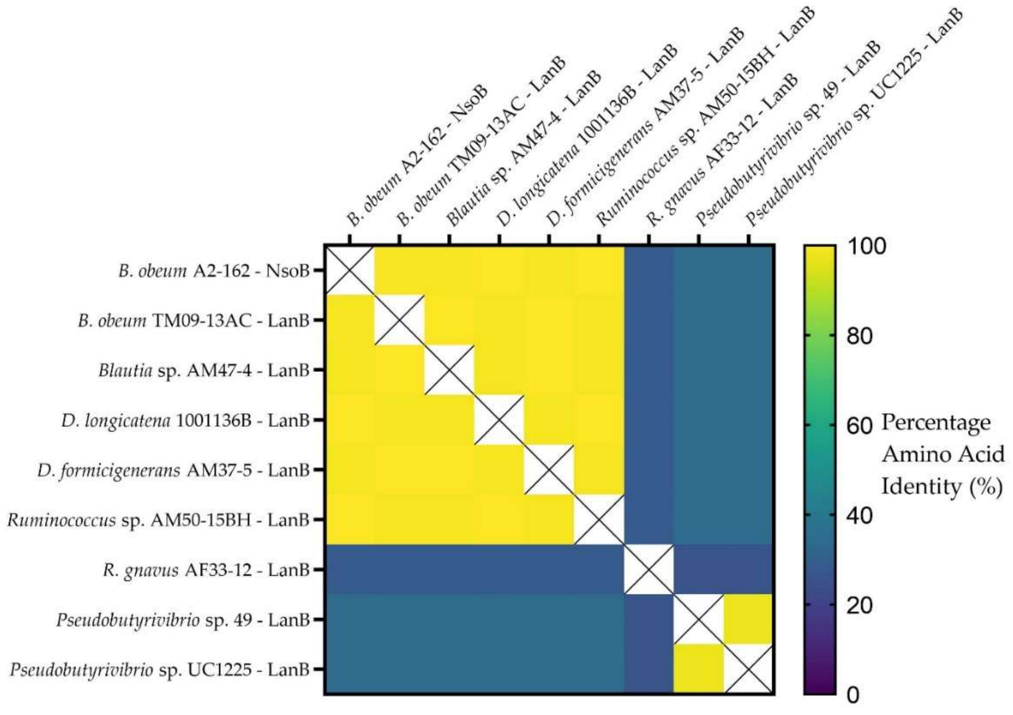

Figure S8. Percentage amino acid identity heatmap of NsoB and LanB from nisin O and nisin O-like clusters.

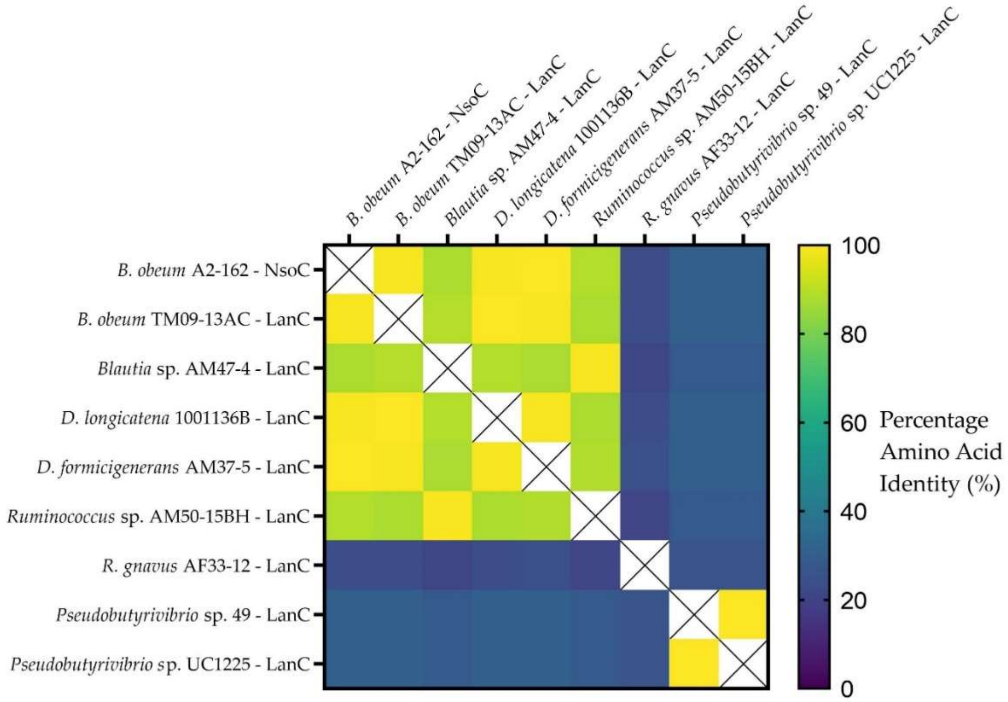

Figure S9. Percentage amino acid identity heatmap of NsoC and LanC from nisin O and nisin O-like clusters.

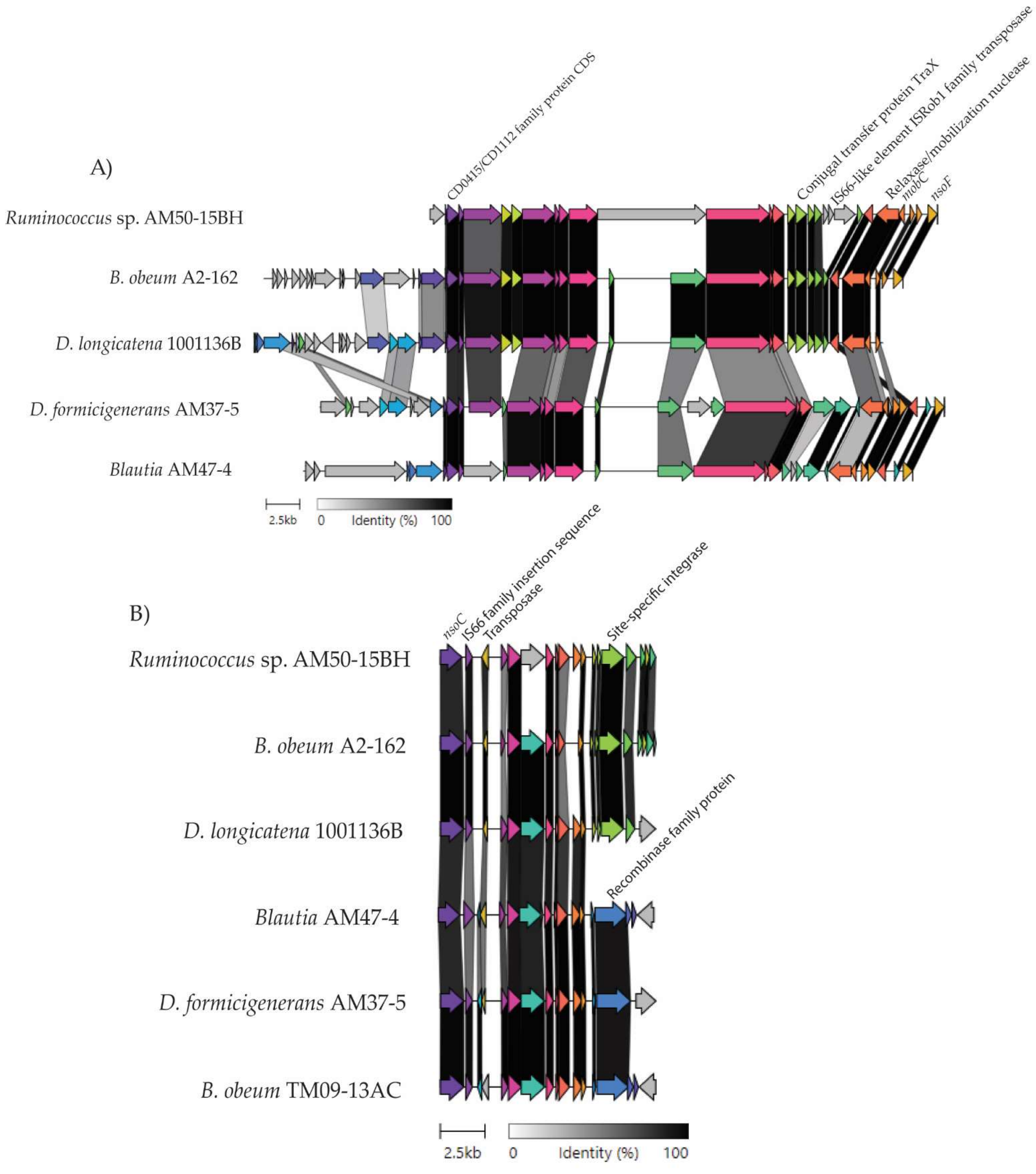

Figure S10 A) Annotated genes 33.3 kb upstream of the start of the nisin clusters, where whole cluster synteny is lost after a CD0415/CD1112 family protein. *B. obeum* AM27-32LB and *Blautia* sp. AM47-4 are not included as the contigs end 5.3 kb and 22.4 kb upstream of the cluster, respectively. B) Annotated genes 7.5 kb downstream of the end of the nisin clusters, where whole cluster synteny is lost after the site-specific integrase/recombinase family gene. *B. obeum* AM27-32LB is not included as the contig ends 3.3 kb downstream of the cluster. The *Blautia* sp. AM47-4 sequence is identical to the other sequences up until the ends of the contig.

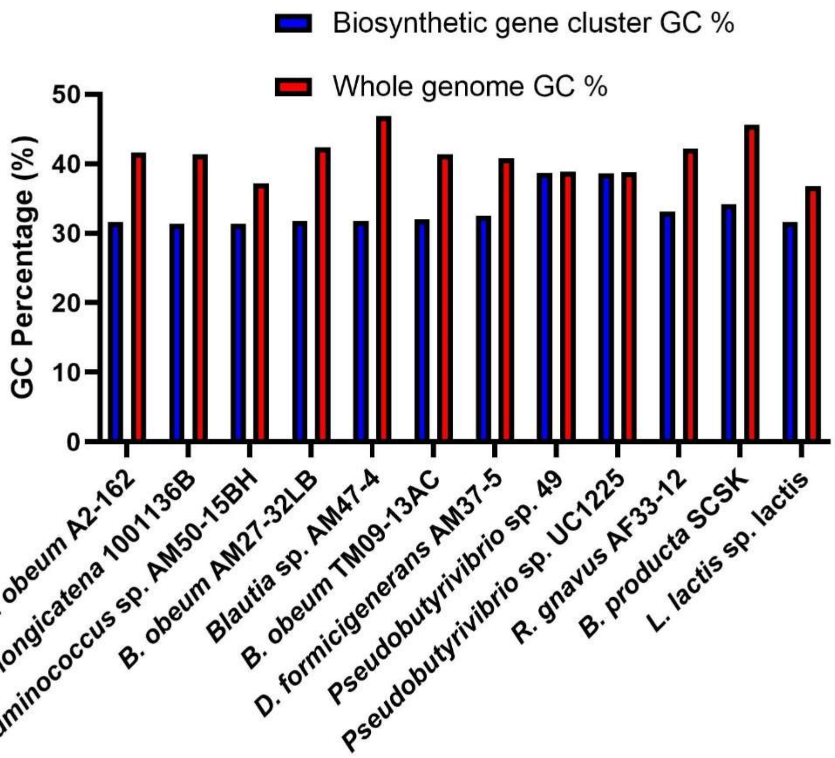

Figure S11. GC content of the nisin O-like clusters (Red) and the whole genomes they are present in (Blue).

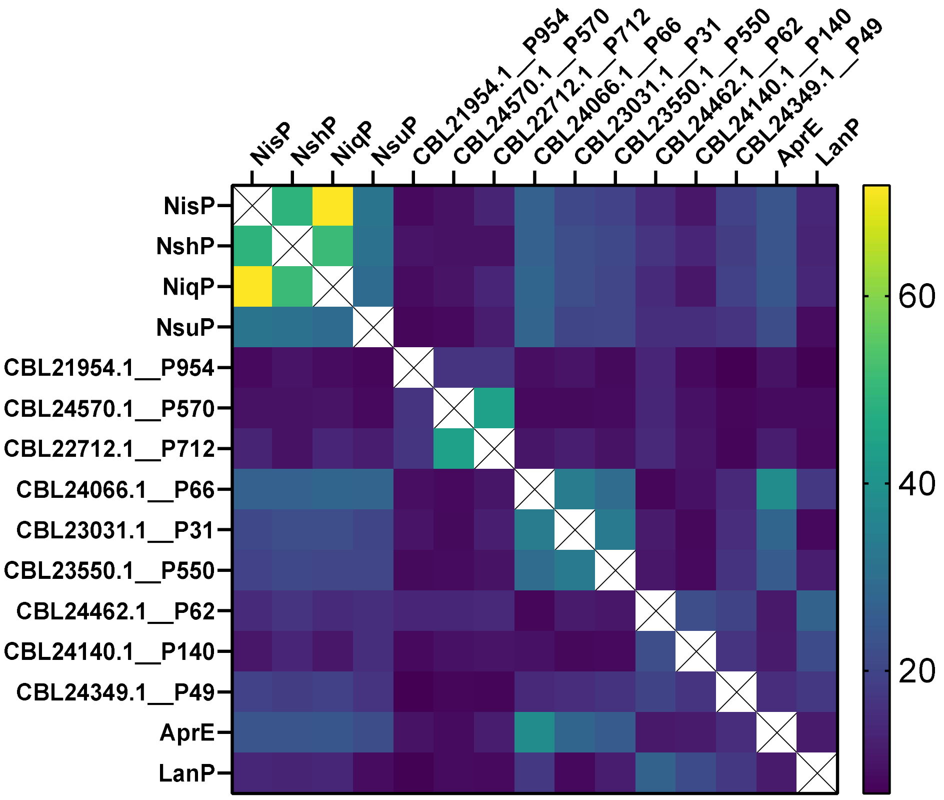

Figure S12. Percentage amino acid identity heatmap of known nisin variant leader peptide cleavage proteins, nine candidate proteases/peptidases from the *B. obeum* A2-162 genome and leader peptide cleaving proteases from subtilin (AprE) and blauticin (LanP) biosynthetic pathways.

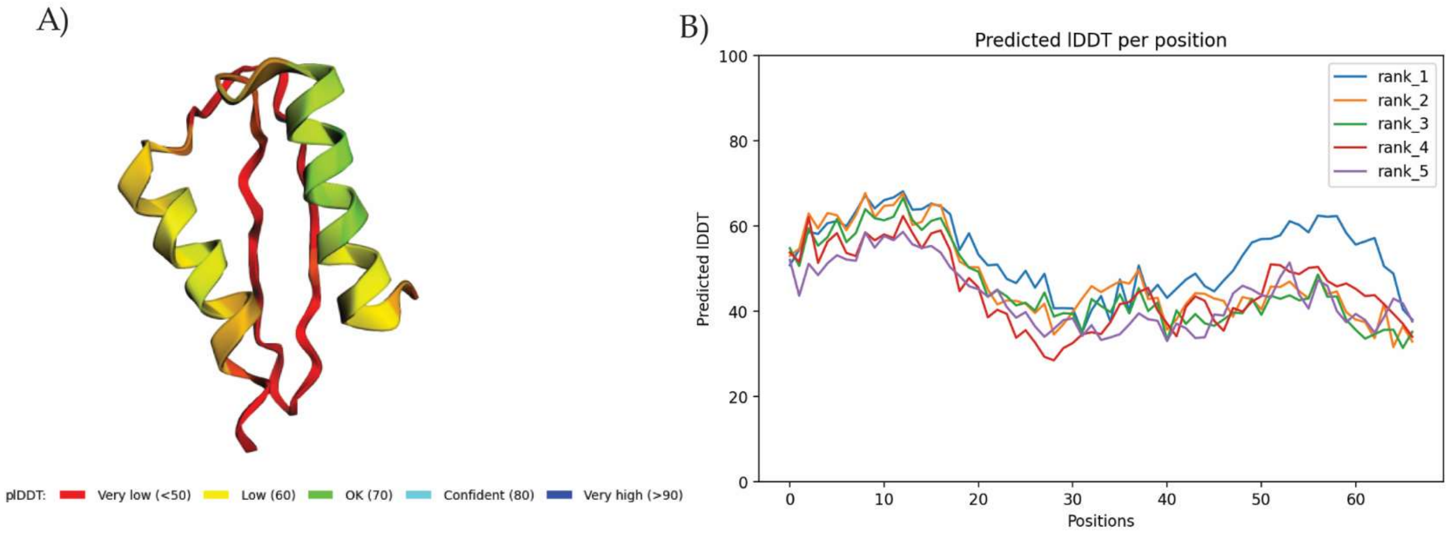

Figure S13. A) Predicted tertiary structure of Nso1.14 from *B. obeum* A2-162. Structure colours represent predicted local distance difference test (plDDT) confidence values. B) plDDT confidence values at each residue in the Nso1.14 sequence.

Table S1. Primers used in this work.

Name	Sequence **(5' -> 3')**
P32_F	TCGAGATCTACCAATTCGGTCCT
P32_R	CTTCCATGGCAAAATTCCTCCGAA
pUK_F (pUK200)	TGAGATAATGCCGACTGTACTT
P32_StuI_F (pUK200)	TCAGATAGGCCTAATGACTGG
P32_NcoI_R (pUK200)	TACCCATGGCAAAATTCCT
P32_R (pUK200)	ATGGCAAAATTCCTCCGAA
P32_BglII_F (pUK200)	CCAAGATCTACCAATTCGGTC
P66NDE_F	GTATACATATGAATCAGCCATTTTATACTGG
P66XHO_R	CTGACTCGAGGATTTCCGTCAATACTTTC
P66CAC_F	AAAAAAATAGCACATGATGACAATGGAC
P66CAC_R	CATTGTCATCATGTGCTATTTTTTTATTT
P570NDE_F	CACCATATGAACGACGAAGAAAAATGG
P570XHO_R	GTAAACTCGAGTTTTTATAAATTCAGTCAGC
P49_NdeI_F	TTTCATATGCGAATTGCGGAAAAAGC
P62_NdeI_F	TTTCATATGAAAGCACTTCGTAATTTTCTGATC
P62_SpliceR_NdeKO	CTCTGAATCATAAGCCGGTGTCGTAT
P62_SpliceF_NdeKO	ATACGACACCGGCTTATGATTCAGAG
pUK200_XhoI_R	TGCACTCGAGCTTAAAGACATTACAC
lanP_NdeI_F	TTTCATATGCGCAAAAAGACCTACCT

lanP_XhoI_R	CAGACTCGAGGTACAGGACGAC
P140_NdeI_F	TTTCATATGGATATAAAAAATTGGAAACAAAGTCC
P140_XhoI_R	ATGACTCGAGTACCACTGTACGTTCA
P32_PstI_F2	GTAAAACGACGGCCAGTGC
P32_SpliceR2K2_R	ACAAGTATATTTTCCATTTCAAAATTCCTCCG
R2K2_SpliceP32_F	AGGAATTTTGAAATGGAAAATATACTTGTGATT
P32_SpliceR1K1_R	ACTAACATCCACCATTTCAAAATTCCTCCG
R1K1_SpliceP32_F	AGGAATTTTGAAATGGTGGATGTTAGTAAAC
p181	GCGAAGATAACAGTGACTCTA

Table S2. Insertion sequences of candidate proteases cloned into the pET15b vector, predicted promoter regions of the nisin O cluster cloned into the pIL253 vector and regulatory sequences cloned into pUK200.

**Vector Backbone**	**Insert**	**Insert Sequence (Nucleotides)**
pET15b	*p66*	ATGAATCAGCCATTTTATACTGGAAAAGGCATCGGAGTGGCAATTCTTGACACAGGAATTTATCCGCATA<br>TAGATTTTGACAGCAGAATCTGTGCTTTTGTGGATTTTATTTTAAATAAAAAAATAGCAcATGATGACAAT<br>GGACATGGAACTTGCGTTGCCGGGATCCTTGCCGGGAGCGGCGCTGCGTCAATGGGAAAATATAAGGGT<br>GCGGCACCGGGCTGTCACCTTGTGGCACTGAAAGTGCTGGATCGCTTTGGCAATGGAAACAAAGAAGAT<br>GTACTGAAGGCATTTGAATGGATCTTATGTAACCGGCAGCGGTATAATATCCGGATCGTGAATATTTCTGT<br>GGGGACAACATATCGGACACGGAGTGAGCAGGATGTACTTGTAAAGGGGGTAGAAAAGCTGTGGGACG<br>AAGGTCTTGTGGTGGTGGCTGCGGCTGGCAATCAGGGGCCGGATCCGGGAAGCGTCACTGCTCCGGGGT<br>GCAGCAAAAAGATCATCACAGTCGGTTCCAGCGACATGCTTTCCGGGAAGCGGGCAGTTTCAGGACGTG<br>GGCCGACGTTTGAGTGTGTGTGTAAACCGGATCTGGTGGCGCCGGGGAAAAACATCATGGCATGTTCGCC<br>CGGAGCGGGCAATCTGTACAGCATGAAAAGCGGCACGTCAATGTCTACTCCGCTGGTTTCCGGGGCAATT<br>GCGCTTGCATTGGAAAAAGATCCGCTGCTTACAAATCTTGAAGTCAAAATGATGTTATGGGAGAGTACG<br>GAGGATATGGGACTGCCGAGAAATCAGCAGGGCTGGGGAAAATTTAACTGTCAAAAATTTCTGGCGCTA<br>TAA
pET15b	*p570*	ATGAACGACGAAGAAAAATGGAATTCGGGAAATGCTCCGCAGAATGATAGAGATACCAAACGGTTCAA<br>TGAACCGCCGAGATATGAACATTATAATATGCATCAGACGTATGTGAATGAAACAGGGCAGGAACCGG<br>AGAAAAAGAAACGCAGAAAGAAAAGCGGTGGCAGGAAACTGGCTTCGACAATTTCTTTTGCGGTAGTGT<br>TCGGACTGGTTGCCGGTGTAGTATTTCAGGGTGTGAATTTTTTTGCAGCTCAGTATATGGGCACAACGACA<br>GATGATGCAGAACCGCAGATTGAGACCGCGCAGCTTGCCGTGAGTGCATCGTCAGATGATGCAGCCTCA<br>GAGGGTACAGATTCTTCTGAGAATGGTACAGATGATGCAGCACAGAGTGTTTCTGCGCAGACAGGAAGT<br>GTTTCAGATGTTGCAAAAGCAGCAATGCCGACAGTTGTCGCAATTACATCTGTCAGCATACAGGAGATTC<br>CGAATTATTTCCGCGCATTTGGATTTGGATATGGCGATACACAGCAGTATTCCAGTGAAGGCAGCGGATC<br>CGGTATCATTGTAGGTGAGAATGATGACGAACTTCTGATCGCAACAAATAATCATGTTGTAGACGGTGCG<br>ACGACACTCAGCGTATGTTTTGCCGGAAGTGATGTAGTCAATGCAGAGGCAGAGACTGTGAATATGTCCT<br>CAGAAAGTGACAGTGATGGAGATGTCAATGTGGAGGATGCCGTCAGTGCGAAGATAAAAGGTACAGAT

		GAAACAAATGATCTGGCGGTAGTTGCGGTACAGAAATCTGATATTCCGGAGGATACCTTATCACAGATC<br>AAGATTGCACAGCTTGGTGATTCTGATTCAGTGGAAGTCGGAGAACAGGTCGTCGCGATCGGTAATGCAC<br>TCGGCTATGGACAGTCTGTAACATCCGGGTGGATCAGTGCATTAAACAGAAGTATTTCGACAAGCGACG<br>GTACGACAAGTGACGGACTGATTCAGACAGATGCAGCAATCAACCCGGGTAACAGCGGCGGCGCACTTC<br>TTAATATGCAGGGAGAAGTGGTTGGTATCAATTCTGCGAAGTATGCAGACAGTGCGGTGGAAGGTATGG<br>GTTATGCAATCCCGATTTCGAAAGCACAGCCGATACTGGAAAATCTTATGAATCGTCAGACGAGAGACA<br>AAGTAGAGGATGACTCAAAAGCAGCATATCTTGGGGTTACTTCTGCCGATCTTTCGATGGAAGCAATCCA<br>GATGTATGATATGCCGGAAGGCGCATTTGTGATCCGTGTCGATAAAGATTCAGCCGCAGGTGAGGCTGGT<br>ATTCAGAAGGGCGATATTATTGTAAGCTTTGACGGCCAGACAGTAAGCGGCAGAGAAGATCTGGAAAAT<br>AAACTGGCTTATTATGAAGCCGGTGAGTCAGTGGATGTTGTTGTTTCCAGAGCTGATAACGGAGAATATG<br>TACAGAAAACAATTTCCGTGACACTTGGAAACCGTTCAGATTACACGGGCTGA
pET15b	*lanP*	ATGCGCAAAAAGACCTACCTGCTGATCGGCAGCATCCAGATCGTCATCATCATCGTCTACATCCTGCTGT<br>TCAAAATCACCATCATCTCCGGCCAAAGTATGAGTCCGACCCTGAAAGATGGTCAGATTACCCTGGTCTA<br>CAAAAGCTGCGACGATTACGAAGTCGGCGAAATCATCACCGTCAACACCAACGAATACGGCGTTTGCGT<br>TAAACGCATTCTGGCAAAAGGCGGCGACGTTATTACCTTCCACGACGGCAAAATCCTGAAAAACGGCAT<br>TGAACTGCAGCCGTACGAATGCGAACCGAATCTGGAACAGGAGTACAACCTGGAAGACGATCAGTACTT<br>CATCATCGGCGACAACTACAAAGCGAGCATCGATAGCCGCAACTACGGTCCGGTCATGAAAAGCGACAT<br>CATCGGCAAAGTCGTCCTGTACC
pET15b	*p140*	ATGGATATAAAAAATTGGAAACAAAGTCCAAAAATTCAGGAAGCAAAAGAAAAACTGGAAGACGAAA<br>AAGTACGCGGTTTTATGCGCTGGGTGTTTGAGATCGTTGTGACACTGGTGCTGGCTGCTATGGTCGGCATT<br>ATGCTGTTTCAGACAGTGACGATGCAGGAAAGTTCGATGGAACCGACGATCGAGGTAGGCGACCGCTTT<br>TTTATAAACCGTGTGGTATATAAATTTACTTCCCCAAAAAGAGGAGATCTGATCGTGTTTCGAACAAATG<br>CCAGTGATGATGCGGCACTGCATATCCGGCGTGTGATCGGACTTCCGGGAGAAACCATCCAGATTTCGGG<br>TGGACGAGTCCTGATCGACGGGGAAGTTTACAAAGAGGGCAAAGATTTTCCTATGATCAGTAATCCGGG<br>ACTTGCGTCCAGTTCTATTACGCTGGAATCCGGTGAATATTTTGTTCTTGGAGATAACAGGAACAACAGT<br>GAAGACAGCCGTTATGCAGATGTCGGTATGATAAAGAAGCGCTATATCGCAGGCAAAATCTGGTTTACG<br>TGTGCACCCTTTGAAAAACTGGGATTTACGAAAGGTTAG
pIL253	*PnsoA1-4*	TCTATTGTGACACAGCATGGTGGAAAAGTAGAATTAAAAAATAAAAGAGGAGCAGAAGTAGATATTATT<br>ATATAGGAAAAATTCATAGTTCAGGTTAGCTCTGGTGGAATACACCAGAGTTTTTTACTTTATAGGATTA<br>GGGTGTCAAAAGCCCATTTTAATATTTGATTTTGCTTGTACCTTGAAAATTTAACACGAATTTGTGAAAGA

		TATAATTTTTGCGCCTATAATTTATTGATATTTTGAAACTGTGGTTCATTGATTTTATGCTGTTTTCTTATTTG<br>AAGCATACGAATCCAAGCTATTGATATAATCTAATAGCTTTTGAAAATCTAATGCTGCTATTTTAAATCCG<br>AAGAATAACCGGGTACGTTTTTTGCCATGCACAGGTATCTTGTCCACATGATATCTTCGACGAAGAAGAG<br>AGGGAATTGCTTCTACTCCATTTCTAAATTTTGCGTATTCAGAATATAGCTAGAAACGACATTGTAAAAA<br>AATATATTAAATTTAGGAACGATTTTGATATTTTGCTGATTTTTATATTGTATAACTAATTTATCAAAAAGA<br>AAGGAGGCACCAA
pIL253	*PnsoBTC*	TGGTGCTGTGGTAAGCATAAAATTACCGATCACATAGAGGAAAAGGGGGAAACCGTAAATTTGGTCATC<br>CCCTTTTCTTACTTTTACATATCCGTGTCACTTTCTAATATAATAAGTAACTCTTCTAATGATGCTTCGTCTA<br>TAAATTTTCCTTCGGGAGTATATGAGTAGCCATATAAGATGGTTCCTTCGTTTTGCAATGACATATGTGTG<br>TACACTAACTTGTCTTTTCCAAATGCTTCAATGTCTTCCTTAACAGAAAGTAGAAGTTCTTCTTTTGTTTTC<br>ATGGAAAGGGCGCCTTTATCTTTTTCTTAAGTATATCATATTTTAGGAATATGTTCAAAAAATCTTGATATT<br>TTTTTGATAATTCCACTTTATAGTTGACTTATAACAGATTAGGAGGTAGAA
pIL253	*PnsoFEG*	TTAGAAGGAACGACCGGCGTATGTCTCTCTCTTCTTGAAGGAGAGAAGAAGAGCGATAATTTATGGAAG<br>AAAGCTTTTTTATTGGCATAAAAAGATAGGATAGGAGTAAACGGTGAATAGTAAGTACTAAGATTTTCGC<br>TAGTTTTCATGAGATAATTACTTTGAAAAATCTAAGATTTTTTAGAGAAATTATCTCATGGATAAAATAAC<br>TCATAAAGTTCGATGTGAACAATGGACAAATATCATCAAAGAATGTCTTGCTAGTGGAATGCCCAAAAT<br>GACTTGGTTCCGAGAGCATGGTATTTCAGATAAATCATTTTTTTACTGGCAACGAATTCTCCGAGAAGAA<br>GCATACCTCTCTACATTAGAGAACACCTTGACGCCAGCTGTTAAAGAAAATTCAGTTCCAACAACTACAG<br>ATTTCGTTGAAATCAAAATGACTGATCACACAAGTTCATCTGCCAGTCCTTTTAAACCGGATGTTGTTATT<br>GAATATCCACATAAAAACAGTGAAGTAAAGCATGTAAATTTGCTATCTGATAAAACTGAGTATGGAATA<br>CAGCCACAAACGGATGGATACTATTATTGGAAAGTCGATAGTAAGACGAATCAGGGAACAACTTATGGT<br>TCATGGAGATTGGGACCTCAGGGTGCCGGCCCAGGTACAGTTTCTGTAAATAAAACAGATACAGTGACT<br>AACACAATTAGTGGATCATATACCAGTGTAGGAGATATATCCGCTTCTTTAGGAGCTACGATTGGGAAGA<br>GCCAGTCCTATTCTGTACAGTACCCTTAATTTCCAGCCTGAGATGCTTTTAGGGCTATTTCAGGCTGGAAA<br>TTAAGGGATGTGCAGGATGTTCTTGAATTATTGTACACTTTAACGCCAGAACAATTAAAAGCGGTGAAAC<br>ATATGATTTCATCCTATCTTGAATTCAAAGAAAATATGGAAGAACAAAATGAGAGATTAAAAAGTGGGA<br>TATCCAATAAATGGATGTCCCCTTTCTGCTTTACATATCAGCAGTCAAAATTATGGAATATTTGTTTATCAT<br>CTTAGCATGTAAGAAAACCGTGAATGTCAACTGATATTGGGGTATTTGTTGAATGCTGCAAATTTGGATA<br>CATTTATGTGGAGAAGGATAGGACCTTACAAGTTCTATCTTTTTTGTTATGCAATTTACAATATAAAAAAT

		TTTTTAATCTTTAGAAAATCTTTATAGTTAATCAGTATACTAAAAAAATCAATTAAAAGGAGATGTATAAT<br>AAT
pIL253	*PnsoR2K2*	TTATACCTGCGAGTATTACAAGTCAACACTCTTTTTGTACCCCCAATTGCCTAACAGGATTTCTTTGTCCAC<br>CAAAAACTCAGCTTACATGCACATGTAAATTAAAGGGTCAATAAATCTGTTTTAGTGTACATACATAAAA<br>AAGCTCTATTTTGATAATAGAGCTTTTTTTACGAGTAAATGAGTATTTTTTACATGTTGGGTGATTAATTAC<br>TAATTTTTGTTATTATTAGTTAGTAAAAACATAAAGGAGCAGATA
pUK200	*PnisA_pepI*	AAACGGCTCTGATTAAATTCTGAAGTTTGTTAGATACAATGATTTCGTTCGAAGGAACTACAAAATAAAT<br>TATAAGGAGGCACTCACCATGCAAATCACAGAAAAATATCTTCCATTTGGAAATTGGCACACCTACTGCC<br>GGATCGTGGGCGAGGCTACTGACCGCGCCCCGCTCCTCCTTCTCCACGGCGGGCCCGGCAGCAGTCACA<br>ACTATTTTGAAGTCCTCGACCAAGTCGCTGAAAAAAGCGGCCGCCAGGTCATCATGTATGACCAATTAGG<br>CTGCGGCAACTCCAGCATCCCCGACGACCAGGCGGAAACGGCCTACACGGCCCAAACCTGGGTCAAGG<br>AGCTGGAAAATGTCAGAGAGCAGCTGGGCCTTGACCAGATCCACCTTTTGGGGCAAAGCTGGGGCGGGA<br>TGCTGGCTTTGATCTACCTGTGCGACTACCAGCCTAAAGGGGTCAAGAGCCTGATCCTCTCCTCCACTTTA<br>GCCTCCGCCAAGCTTTGGAGCCAGGAACTGCACCGCTTGATCAAGTACCTGCCCAAGGGCGAGCAGGCC<br>GCTATCAAGGAAGCTGAAACAACTGGCAACTACGATTCCCCGGCCTACCAGGCGGCCAATGCCCACTTC<br>ATGGACCAGCACGCCATCAAGCTTACGCCGGACCTGCCGGAGCCAGTTTTGCGCAAAAAAAAGGGCGG<br>CAACCTGGCCTACTTGACAGGCTGGGGCCCTAATGAATATACGCCGATTGGCAACCTGCACGGCTATGA<br>GTACACTGACCGCTTAAAGGACCTGGACTTGCCGGCCTTGATTACCAGCGGCACTGACGACTTGTGCACT<br>CCCCTAGTGGCTAAAAGCATGTACGATCACTTGCCAAATGCCCGCTGGGAGCTCTTTGCCGGCTGCGGCC<br>ACATGCCTTTTGTCCAGGAAAATGCCAAGTATCAAGAGCTGTTGTCTGACTGGTTAA
pUK200	*P32_R1K1*	GATATGATAAGATTAATAGTTTTAGCTATTAATCTTTTTTTATTTTTATTTAAGAATGGCTTAATAAAGCGG<br>TTACTTTGGATTTTTGTGAGCTTGGACTAGAAAAAAACTTCACAAAATGCTATACTAGGTAGGTAAAAAA<br>ATATTCGGAGGAATTTTGCCATGGTGGATGTTAGTAAACTGCGGCAATATAATTTGTTGATTTTGGATGTT<br>ATGATGCCGGATATTGATGGTTTCACATTTTGCAAAAAAATCCGTGACATATTCAATGCACCAATTTTATT<br>TCTGACAGCTAAAACACTGGAAGACGAAGTATTATATGGTTTAGGTATTGGGGGAGACGATTATATTACA<br>AAACCCTTCCGGATTAAGGAGTTAAGAGCTCGTGTAAAAGCACACTTAAGGCGAGAGTCCAGAGAAAAT<br>CATGATTATTTATTTTCAGAAGATGGAAATATTAAGTTTGATTTGTCAGCAAAAAAGCTATTGGTTTGTGA<br>AAAGGAAATTCCTTTGACACATAGCGAATACAAAATATGTGAATTGCTGATACAGAATAGAGGACACGT<br>TTTTTCCAGAGAGAAAATATATGAACGAGTTTTTGGATTTTATGGAGAATCAGCAGATAATACAATAGTT<br>GTTCATGTTAAAAATATACGAGTGAAACTAAGTGAAGCTGGTGTGAATCCTATACAGACAGTGTGGGGG

		ATAGGATATAAATGGGAGTAAAAATAAAAAAAAGGCAGTATTCTCTAAAGTTTTTTTTATAAAGTTTAT<br>ATTGTCATTGATTATAGGAGCTGGAGTGAGTATTGCCTTACCGCTGGTCTTGGCAACACTTGCAAGTAATA<br>TGGGATATATAACTGTTGCAAATTATAATGAGATACAGGCTGAGAAAACGGCAAAAATTTTAGAAACAG<br>AAAAAAATCCTGACTATAAGAATATTCCAGCAGGGATAAAATACTTAATAATTAGCAAAGAATTCGATA<br>TCTTAAATACTAATATGAGCAATGGGGAACAAAAAGACGCATTGAGATATGCAAATGGTAAGTTTGAGA<br>AAACAGCATCAGGTAAACAATTTATTTTAGTTACAAGAGATAAAGAATTCTGTATTTTACAATATTATATT<br>GGTTCTCATTTCACCAATATATGGTTAGATATACATATGCCATCTCCAGATATTTTGATTAATGCAGGAAT<br>GATTTTAAATTGTTTATTTGTATTTAGTATTATGGTCTTTTTGTTTGCAAAAGAACTAAGAAAAGAGTTGAA<br>GCCAGTAATGGATGCGACTACAAAAATAGAAGAGCAGGAGCTGGAATTTAATATTTCATCATCGAGGAT<br>AATAGAATTTAATGATATATTAAAATCTATTTATAATATGAAAAATAGTTTAAAAAAGTCTTTGAAAACG<br>CAATGGAATATAGAGTGTCAGCAGAAAGAACAAATTTCGGCATTAGCGCACGATTTGAAGACACCGTTG<br>ACGATCATAGGTGGTAATGCTGATTTATTATCTGAAACAAATATTAATCAGGAGCAAGAAGAGTATATTA<br>ACCATATTTTAGAGAGCTCTAAACGCATGGAAAATTACATTGCTATATTGATTGATCTATCCAAGAATAC<br>TGGTGAGATTCCGATAAATAGAGAAAGCATATCTATTGGAAAGTTTATTGATAGTATAAAAGGACAGAT<br>GCAGTCTGTAGTTGCAATGAAAAAAATGAATTTAATAATTGCAATTATGGATGAATCTTTTAGGATAGAG<br>ATTGATACAGTATTAATGGAAAGGGCGATTATAAATGTTTTATCAAATGCCGTAGATCATTCTCCTACAC<br>ATGGGGAGATTATTTTTAACGTGACGAAGGATAAAGGAAAATGTAAGATTAGTATTATAGATTCAGGTCC<br>TGGTTTTACGCCGGCAGCATTAAAATATGGTTTAGAAAGATTTTTTATGGATGATAAGAGTAGAAATTAT<br>CAACACCATTATGGAATGGGGTTATATATTACAAATTCTATTGTGACACAGCATGGTGGAAAAGTAGAAT<br>TAAAAAATAAAAGAGGAGCAGAAGTAGATATTATTATATAG
pUK200	*P32_R2K2*	GATATGATAAGATTAATAGTTTTAGCTATTAATCTTTTTTTATTTTTATTTAAGAATGGCTTAATAAAGCGG<br>TTACTTTGGATTTTTGTGAGCTTGGACTAGAAAAAAACTTCACAAAATGCTATACTAGGTAGGTAAAAAA<br>ATATTCGGAGGAATTTTGCCATGGAAAATATACTTGTGATTGACGATGACGAAAAGATTTTAGAATTAGT<br>ACAAGAAGTTTTGAAAAATGAACATTATATAGTGGAAACAAGAAGCTATGTTGACAATACTAATATTGG<br>TGAATTTGAAGGGTTTGACTTAATATTATTGGATATTATGTTACCGTTTTTAGATGGTTATGAAATTTTGGA<br>GAGAATAAAAAACATAATAACATGTCCGGTGATCTTTTTATCTGCTAAGTCTAGTGAAGGAGCAAAAGT<br>GAAAGGCCTGATGAGTGGAGCAGATGATTATATTACCAAACCTTTTAGTATCAGAGAGTTAGTAGCCAG<br>AGTGAAAGTTGCATTAAGAAGAAATTTGAAAAATGGGAGTAACGGATTTTAATCAATGGATTGGTGTTT<br>AATCAAGATTCAAACTCAATAAAATTAGATAATGGTGAAATTTTATTAACGAAAAATGAGTTTCGCATAT<br>GTAAAATTCTTGTTCAGAATAGCGGAAAGATTTTTTCAAAAGATGAGCTATATGAATACTTATATGACTT

		AGATGCCAATGCACAATTAAGGACTATAACAGAGTTTATTTACTCAATCAGAAAAAAGTTCAAAACACTTGGTCTTGATCCAATAAAAACTATTTGGGGGATAGGTTATAAATGGGATATAAAATAATAACGATGAAGAGACAGTTATTACTGTACTTGATAAAATTGTTTATGGGAATCGTGATTATAATAATCTGTTGGTTGGCTTTAACTAATATATTTGTGGAATGCGGAGTGATAATTCCAGCAAATTATAGCGAAACTATTTTGGAAGAGAATAGAAAGCGTTTAGATGATATACAACAGATAACAGATAATGATTTACCATATGGAAGTAAGTATTCAATCTTTGATTTAGATTATAATTATGAACGTGGGACTATGAATAAATCAGATATAGAAGTAACTAAAAAAAATATTATTAGGGAAAGAAAGTAATCTTCAAGGGAATTATGTTTACTCAGTAATTGCTAGAACAGAAGAATATTGCGTTATAAAGTATAATATTAAAGCACGTTTTAACTCTGACAATAAAATTTTCAATTATATAGATTATGACAATTTGTCGTATATCACAATGGTATTAGTGTTTTTACTATATGTGTATATTATGACATTACACTTAGTTGGCATCTGGAAAAATAATTTTGAAAAGATAGAGAAAATTACGCTGGAAATAGAAAAACAGAATCTGGATTTCACTTACGAAGAAAGTAAGATAAAAGAATTTTCTAATATTATTACAGCATTAATTAAGATGAGGGATGCATTAAAAGAGTCATTGTATCAAACCTGGAAGATAGAAAATGAAAAAAATGAGGAAATAGCAGCATTGGCTCATGATATAAAAATACCGCTTACAATTATCAGTGGAAATACAGAGCTTTTAAAATGTTATTCATCTGATGAGTACAGTTTATCTCATCTGCAAAGTATTTGGGGAGCTGTGGGCAAGATGGAGGAATACATTAATTTATTAATTAAATATGTGAAAGCTGATAGAATTGATTTTCACGAAAAGGAAAGTATGCCCTGCAACAGTTTTTCTCATAAAATAGTCTCTGAGATTAAAGAGTATATCAGCGGATCTGAAGAAATAATTAAATTTAATGTTGAAAAAGTGAGGGGCACTATTAAGATAGACTACATATCTTTAGAAAGGGCAATTTTTAACATTATTGATAACGCAATAGAATATAAGGTATATGGTGACAAAATAATGTGTTTTATTGGAAAAAAAGATGATTCTTATATATTTACCATATGTAATGAAAAAGGAGAGTTTAGTAGTCAGGTTCTTGAGAATGGAACTAAATTATTTTTTACTTCAAATAATAATAGAAATTCAATTCACTATGGTATTGGCCTAGCTTATGCGAATAAAGTTATTCAATCTTGTGAAGGGGAATTGGAACTATACAATTCTAAAGAGCATGGTGCTGTGGTAAGCATAAAATTACCGATCACATAG
pUK200	*P32_nso1.14*	GATATGATAAGATTAATAGTTTTAGCTATTAATCTTTTTTTATTTTTATTTAAGAATGGCTTAATAAAGCGGTTACTTTGGATTTTTGTGAGCTTGGACTAGAAAAAAACTTCACAAAATGCTATACTAGGTAGGTAAAAAAATATTCGGAGGAATTTTGCCATGAAAACAAAAGAAGAACTTCTACTTTCTGTTAAGGAAGACATTGAAGCATTTGGAAAAGACAAGTTAGTGTACACACATATGTCATTGCAAAACGAAGGAACCATCTTATATGGCTACTCATATACTCCCGAAGGAAAATTTATAGACGAAGCATCATTAGAAGAGTTACTTATTATATTAGAAAGTGACACGGATATGTAA

Table S3. Analysis of the 59 Lachnospiraceae genomes that contained either *nisB* and/or *nisC*. The presence or absence of the *nisB* or *nisC* genes, the bacteriocin biosynthetic gene cluster, presence or absence of immunity genes and isolation source are displayed. Genomes which contained *nisB*, *nisC* and four putative structural peptides within the biosynthetic gene cluster are highlighted.

**Bacteria**	**Strain**	***nisC*-like gene/s**	***nisB*-like gene/s**	**Structural peptides**	**Lantibiotic Immunity Genes**	**Isolation Source**
uncultured *Butyrivibrio* sp.	UMGS920	0	1	Rathipeptide, Thiopeptide, LAP, Zoocin A	No	Human gut
uncultured *Lachnospiraceae* bacterium	UMGS1368	0	1	Zoocin A	No	Human gut
Luxibacter massiliensis	Marseille-P5551	0	1	Number of potential clusters and structural peptides	Yes	Human stool
Dorea formicigenerans	AM42-8	2	1	LanB present but no structural peptides	Yes	Human faeces
Dorea formicigenerans	**AM37-5**	1	1	4 - Lanthipeptide	Yes	Human faeces
Blautia obeum	**AM27-32LB**	2	2	4 - Lanthipeptide	Yes	Human faeces
Dorea longicatena	AM23-13	1	0	Plantaricin A family structural peptide (LanM)	Yes	Human faeces
[Eubacterium] rectale	AF39-14AC	0	2	No cluster present	Yes	Human faeces
Eubacterium rectale	AF38-24	0	1	1 - Class IIb bacteriocin (lactobin A/cerein 7B family)	Yes	Human faeces
Ruminococcus gnavus	**AF33-12**	4	2	4 - Lanthipeptide	Yes	Human faeces
Clostridium sp.	AF37-7	1	0	No cluster present	No	Human faeces
Clostridium sp.	AF36-18BH	0	1	No cluster present	Yes	Human faeces
Clostridium sp.	AF34-10BH	0	1	Lasso peptide biosynthesis machinery	Yes	Human faeces
Firmicutes bacterium	AM59-13	0	1	Colicin, Zoocin A	No	Human faeces
***Ruminococcus* sp.**	**AM50-15BH**	2	1	3 - Lanthipeptide	Yes	Human faeces
Firmicutes bacterium	AF22-6AC	0	1	Zoocin A	Yes	Human faeces
***Blautia* sp.**	**AM47-4**	2	1	4 - Lanthipeptide	Yes	Human faeces
Clostridium sp.	AM43-3BH	1	1	1 structural peptide and LanB	No	Human faeces
Firmicutes bacterium	AM43-11BH	0	1	Ranthipeptide (exopolysaccharide), NRPS (Dipeptide Aldehydes)	Yes	Human faeces
Clostridium sp.	AM34-9AC	0	2	Zoocin A, Lasso peptide	Yes	Human faeces
Blautia obeum	**A2-162**	1	2	4 - Lanthipeptide	Yes	Human faeces
Dorea formicigenerans	4_6_53AFAA	3	1	LanB present but no structural peptides	Yes	Gastrointestinal tract

Lachnospiraceae bacterium MD308	03_02	3	1	1 structural class I lanthipeptide	Yes	Mouse ceca
Dorea longicatena	AGR2136	1	0	Plantaricin A family structural peptide (lanM) and BlpK-like bacteriocin cluster	Yes	Rumen
Clostridium sp.	TF08-15	0	1	Lasso Peptide Biosynthesis Machinery, Ranthipeptide, Zoocin A	Yes	Human faeces
Lachnospiraceae bacterium	GAM79	1	2	1 structural class I lanthipeptide, lasso peptide	No	Human faeces
Butyrivibrio sp.	X503	1	0	7 - Type II lanthipeptide (LanM)	No	Buffalo rumen
Clostridium sp.	E02	1	1	1 structural class I lanthipeptide, 1 class II lanthipeptide	Yes	River Sediment
Dorea longicatena	1001175st1_H1	2	1	No cluster present	Yes	Human stool
Dorea longicatena	P3wC11	1	1	No cluster present	No	Human faeces
uncultured Clostridium sp.	2789STDY5834873	0	1	Lasso peptide	No	Human faeces
Clostridium sp.	Marseille-P2538	1	1	1 structural class I lanthipeptide, 1 class II lanthipeptide	Yes	Human faeces
***Pseudobutyrivibrio* sp.**	**49**	1	1	4 - Lanthipeptide	Yes	Rumen
***Pseudobutyrivibrio* sp.**	**UC1225**	1	1	4 - Lanthipeptide	Yes	Rumen
Dorea sp.	Marseille-P4042	2	1	1 structural class I lanthipeptide	Yes	Human stool
uncultured Clostridiales bacterium	UMGS361	0	1	2 class II lanthipeptides (LanM) and 2 structural gallidermin like structural genes	No	Human gut
Clostridium indicum (Firmicutes)	PI-S10-A1B	1	1	1 structural class I lanthipeptide, 2 class II lanthipeptide (lanM)	Yes	Industrial effluent plant sludge
Clostridium sp.	AM25-23AC	1	0	UviB like structural peptides	No	Human faeces
Clostridium sp. (Firmicutes)	AF28-12	1	1	No cluster present	No	Human faeces
Clostridiaceae bacterium (Firmicutes)	AF02-42	1	1	Ranthipeptide	No	Human faeces
Clostridiaceae bacterium (Firmicutes)	TF01-6	2	1	Partial cluster, no structural genes	No	Human faeces
***Blautia obeum* (Firmicutes)**	**TM09-13AC**	1	1	4 - Lanthipeptide	Yes	Human faeces
Dorea formicigenerans	TF09-3	0	1	No cluster present	Yes	Human faeces
Ruminococcus gnavus (Firmicutes)	AF27-4BH	3	1	3 class II lantibiotic	Yes	Human faeces
Eubacterium rectale	AF25-15	0	1	No cluster present	Yes	Human faeces
Dorea longicatena	AF17-8AC	1	0	No cluster present (2 class I structural genes)	Yes	Human faeces

[Eubacterium] rectale	AF17-27	0	1	3 - bacteriocin structural genes	Yes	Human faeces
Ruminococcus sp. (Firmicutes)	AF32-2AC	1	1	1 class I lanthipeptide	Yes	Human faeces
Ruminococcus sp. (Firmicutes)	AM49-8	1	1	1 class I lanthipeptide	Yes	Human faeces
Ruminococcus sp. (Firmicutes)	AM49-10BH	1	1	1 class I lanthipeptide	Yes	Human faeces
Ruminococcus sp.	OM04-4AA	0	2	1 class I lanthipeptide	Yes	Human faeces
[Ruminococcus] gnavus (Firmicutes)	AF13-14A	3	1	1 class I lanthipeptide	Yes	Human faeces
[Eubacterium] rectale	2789STDY5834884	0	1	Bacteriocin cluster - no structural peptide	No	Human faeces
Blautia sp. (Firmicutes)	YL58	1	1	5 - Lanthipeptide	Yes	Mouse ceca
Merdimonas faecis (Firmicutes)	BR31	2	1	2 class I lanthipeptide (1 with lanM)	Yes	Human stool
Drancourtella sp. (Firmicutes)	An177	1	1	1 class I lanthipeptide	No	Chicken caecum
Blautia coccoides (Firmicutes)	YL58	1	1	5 - Lanthipeptide	Yes	Mouse ceca
Hungatella sp. (Firmicutes)	UBA3048	1	1	1 class I lanthipeptide	No	Wood
Hungatella xylanolytica (Firmicutes)	DSM 3808	1	1	1 class I lanthipeptide	Yes	Cattle manure

Table S4. Candidate proteases identified through a manual genome search of the *B. obeum* A2-162 genome using query terms 'subtilisin' and 'serine-like'.

Geneious ID	Length (bp)	Protein ID	% Similarity with NisP
Trypsin-like serine proteases, typically periplasmic, contain C-terminal PDZ domain coding sequence (CDS) (P712)	1,509	CBL22712.1	14.8
Trypsin-like serine proteases, typically periplasmic, contain C-terminal PDZ domain CDS (P570)	1,509	CBL24570.1	14.6
Trypsin-like serine proteases, typically periplasmic, contain C-terminal PDZ domain CDS (P954)	1,332	CBL21954.1	12.7
Subtilisin-like serine proteases CDS (P31)	1,722	CBL23031.1	16.1
Subtilisin-like serine proteases CDS (P66)	837	CBL24066.1	18.0
Subtilase family CDS (P550)	1,689	CBL23550.1	13.8

Table S5. Candidate proteases identified through a tBLASTn search of the *B. obeum* A2-162 genome using the amino acid sequence of LanP (*B. producta* SCSK) as the query sequence. P140 was identified through analysis of LanP conserved domain 26_SPase_1 in proteins within the *B. obeum* A2-162 genome.

Geneious ID	Length (bp)	Protein ID	% Similarity with LanP
Signal peptidase I, bacterial type CDS (P62)	486	CBL24462.1	25.6
Signal peptidase I archael type CDS (P49)	486	CBL24349.1	17.8
Signal peptidase I, bacterial type CDS (P140)	594	CBL24140.1	23.8
